# *Plasmodium* P-Type Cyclin CYC3 Modulates Endomitotic Growth during Oocyst Development in Mosquitoes

**DOI:** 10.1371/journal.ppat.1005273

**Published:** 2015-11-13

**Authors:** Magali Roques, Richard J. Wall, Alexander P. Douglass, Abhinay Ramaprasad, David J. P. Ferguson, Mbinda L. Kaindama, Lorenzo Brusini, Nimitray Joshi, Zineb Rchiad, Declan Brady, David S. Guttery, Sally P. Wheatley, Hiroyuki Yamano, Anthony A. Holder, Arnab Pain, Bill Wickstead, Rita Tewari

**Affiliations:** 1 School of Life Sciences, Queens Medical Centre, University of Nottingham, Nottingham, United Kingdom; 2 Biological and Environmental Sciences and Engineering (BESE) Division, King Abdullah University of Science and Technology, Thuwal, Kingdom of Saudi Arabia; 3 Nuffield Department of Clinical Laboratory Science, University of Oxford, John Radcliffe Hospital, Oxford, United Kingdom; 4 UCL Cancer Institute, University College London, London, United Kingdom; 5 Mill Hill Laboratory, The Francis Crick Institute, Mill Hill, London, United Kingdom; Albert Einstein College of Medicine, UNITED STATES

## Abstract

Cell-cycle progression and cell division in eukaryotes are governed in part by the cyclin family and their regulation of cyclin-dependent kinases (CDKs). Cyclins are very well characterised in model systems such as yeast and human cells, but surprisingly little is known about their number and role in *Plasmodium*, the unicellular protozoan parasite that causes malaria. Malaria parasite cell division and proliferation differs from that of many eukaryotes. During its life cycle it undergoes two types of mitosis: endomitosis in asexual stages and an extremely rapid mitotic process during male gametogenesis. Both schizogony (producing merozoites) in host liver and red blood cells, and sporogony (producing sporozoites) in the mosquito vector, are endomitotic with repeated nuclear replication, without chromosome condensation, before cell division. The role of specific cyclins during *Plasmodium* cell proliferation was unknown. We show here that the *Plasmodium* genome contains only three cyclin genes, representing an unusual repertoire of cyclin classes. Expression and reverse genetic analyses of the single Plant (P)-type cyclin, CYC3, in the rodent malaria parasite, *Plasmodium berghei*, revealed a cytoplasmic and nuclear location of the GFP-tagged protein throughout the lifecycle. Deletion of *cyc3* resulted in defects in size, number and growth of oocysts, with abnormalities in budding and sporozoite formation. Furthermore, global transcript analysis of the *cyc3*-deleted and wild type parasites at gametocyte and ookinete stages identified differentially expressed genes required for signalling, invasion and oocyst development. Collectively these data suggest that *cyc3* modulates oocyst endomitotic development in *Plasmodium berghei*.

## Author Summary

The malaria parasite is a single-celled organism that multiplies asexually in a non-canonical way in both vertebrate host and mosquito vector. In the mosquito midgut, atypical cell division occurs in oocysts, where repeated nuclear division (endomitosis) precedes cell division, which then gives rise to many sporozoites in a process known as sporogony. The molecular mechanisms controlling this process are poorly understood. In many model organisms including mouse and yeast cells the cell cycle is regulated by members of the cyclin protein family, but the role of this family in the malaria parasite is unknown. Here, we show that there are only three cyclin genes and investigate the function of the single P-type cyclin (CYC3) in the rodent malaria parasite, *Plasmodium berghei*. We show that CYC3 has a cytoplasmic and nuclear localisation throughout most of the parasite lifecycle and by gene deletion we demonstrate that CYC3 is important for normal oocyst development, maturation and sporozoite formation. Moreover, we show that deletion of *cyc3* affects the transcription of genes required for cell signalling and oocyst development. The data suggest that CYC3 modulates asexual multiplication in oocysts and plays a vital role in parasite development in the mosquito.

## Introduction

The mechanisms of mitotic cell division and the various molecules involved are well studied in many model systems including yeast, plants and human cells. Progression through mitosis is controlled by a range of factors, including cyclins, protein kinases (PKs) and phosphatases (PPs), and the anaphase-promoting complex (APC) components [1–4]. Cyclins play active roles at distinct stages of the cell cycle [4] via regulation of cyclin-dependent kinases (CDKs). Cyclins possess a conserved ~100-residue sequence known as the cyclin box that mediates CDK binding and activation [5]. Certain cyclins are capable of binding several CDKs, which are themselves able to associate with multiple cyclins [6,7]. These distinct but overlapping functions orchestrate cell cycle progression.

In most model systems, the synthesis and level of cyclins are tightly regulated during the cell cycle, with each cyclin being degraded via ubiquitination once its function is complete [8,9]. Key cell cycle transitions are regulated by specific cyclins: G1/S cyclins, which are essential for cell cycle entry at G1/S (start), and G2/M cyclins, which are essential at the G2/M (mitosis) transition. In some species, there are multiple forms of G1 and G2 cyclins. For example, in vertebrates there are at least three G1 cyclins (C, D, and E) and two G2 cyclins (A, which is also active in S phase, and B). Many other cyclins with additional functions have also been described [10,11].

Recent bioinformatic analyses have identified 3 distinct classes of cyclin: Group I (including cyclin A, B, D, E, F, G, I, J, and O families), Group II (P/Pho80-like, and Y), and Group III (C, H, K, L, and T) [12,13]. Group I includes known essential cell cycle cyclins including some involved in mitosis and meiosis [10,14,15], while Group III contains cyclins associated with transcription and RNA processing [11]. Group II contains both cell cycle cyclins and those of other function, such as cell metabolism (e.g. P-type cyclins (CYCPs), which were originally believed to be unique to plants) and appear to link cell cycle regulation to nutritional status [16–18]. The cyclin box of CYCP in plants shows high similarity to the corresponding domain of trypanosome cyclins CYC2, CYC4, CYC7, CYC10 and CYC11 [13,19] and *S*. *cerevisiae* PHO80-like cyclins [20].

Malaria parasites, *Plasmodium*, which belong to the phylum *Apicomplexa*, divide and proliferate in an unusual way compared to other eukaryotes. During its life cycle, *Plasmodium* exhibits two types of cell division; one in asexual stages that resembles endomitosis and one in sexual stages. The endomitotic-like asexual stage is characterized by multiple nuclear divisions preceding cytokinesis, with maintenance of the nuclear membrane wherein the microtubule organising centre (MTOC) or spindle body is embedded [21–23]. This process is observed at three developmental stages of the parasite life cycle: during schizogony, replication and multiplication within liver and red blood cells in the vertebrate host [21], and at the sporogonic stage of parasite development in the mosquito vector [24]. The other type of cell division occurs during male gametogenesis, where three rounds of rapid DNA replication are followed by cell division and chromosome condensation giving rise to eight microgametes [23,25,26].

In the human malaria parasite, *Plasmodium falciparum*, the stages of development during asexual multiplication inside red blood cells have been described as (a) ring stage (early trophozoite) with a single (haploid) interphase nucleus in G0, (b) mature trophozoites ready for chromosome replication (G1) and undergoing DNA synthesis (S phase), and (c) the schizont stage when asynchronous nuclear division begins (M phase) and repeated S and M phases continue resulting in a multinucleate syncytium [21,27]. At the end of the growth phase, cell division occurs in the late stage schizont or segmenter to form merozoites that are released to invade new red blood cells. This process resembles that of endomitosis observed in *Drosophila* cells [28].

The molecular mechanisms that regulate nuclear and cell division in the malaria parasite remain largely unknown. We have previously shown that the single putative homologue of cell division cycle protein 20 (CDC20), a well-characterised activator of the anaphase promoting complex/cyclosome (APC/C), is crucial for karyokinesis and cytokinetic control of male gametogenesis [29]. Furthermore, systematic genome-wide functional analysis of the protein kinome and phosphatome has identified molecules crucial for both male gametogenesis and asexual multiplication during sporogony, including CDKs, CDPK4, MAP2 and PTPLA [30–32]. *Plasmodium* has a number of genes that code for calcium dependent protein kinases (CDPKs) that are implicated in control of growth and cell division [31,32]. Other kinases, such as ARK1, an aurora-like kinase associated with the spindle pole body, have been identified and implicated in cell division [33]. Knowledge of cyclin function in *Plasmodium* is limited. Previously, four cyclin genes were described in *P*. *falciparum*, *Pfcyc1* to *-4* [34,35]. Biochemical studies showed that *Pf*CYC1, *Pf*CYC3 and *Pf*CYC4 associate with histone H1 kinase activity present in the parasite extract [35], and *Pf*CYC1 and *Pf*CYC3 bind and activate the *Plasmodium* CDK1 homologue (PK5) *in vitro* [34,35]. *Pf*PK5 has also been shown to be activated by mammalian proteins (p25 and RINGO) that have no detectable primary sequence similarity to cyclin [34,35]. Recently, *Pb*CYC3 has been shown to be a target gene of the AP2-O transcription factor, and is involved in oocyst development [36].

Here, we describe an in-depth phylogenetic analysis of the cyclin repertoire in *Plasmodium*. We then focus on the single P-type cyclin (CYC3) and examine its role during parasite cell division, using the rodent malaria model *Plasmodium berghei*. We show that whilst CYC3 is dispensable for asexual cell cycle progression in the mammalian host, it modulates oocyst development and the subsequent differentiation of sporozoites, the endomitotic process of sporogony within the mosquito vector.

## Results

### *Plasmodium* parasites have a small, unusual complement of cyclins

Cyclins are a diverse superfamily of proteins (for example see [13]). A number of PFam models incorporate parts of the conserved domain (including PF08613, PF00134, PF02984), but none covers the entire conserved region and each is biased towards particular classes.

**Fig 1:**
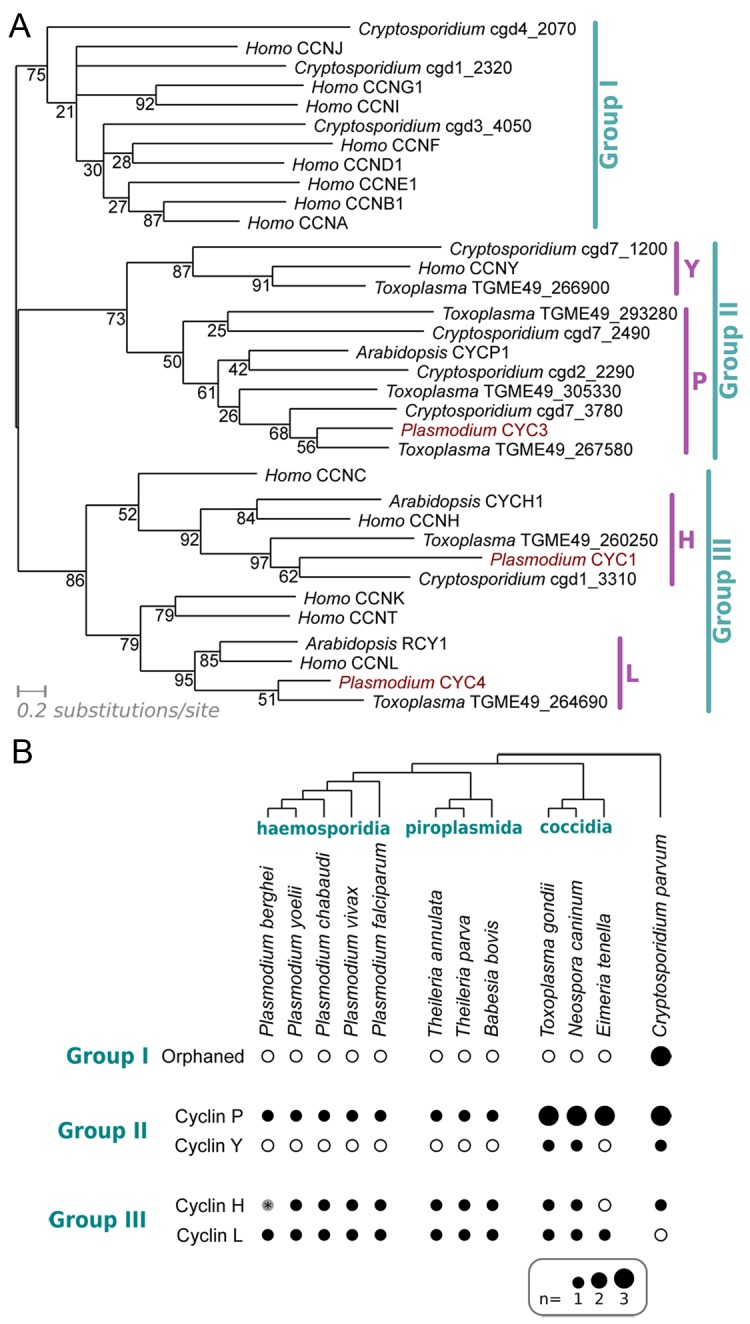
Characterisation of the cyclin repertoire of the *Apicomplexa*. (A) Maximum likelihood phylogeny based on an alignment of cyclins from *Plasmodium falciparum*, *Toxoplasma gondii*, *Cryptosporidium parvum* and *Homo sapiens*. CYCP1 and CYCH1 from *Arabidopsis thaliana* have been included for clarity. Topology support from bootstrapping is shown at nodes. (B) Distribution of cyclin families across *Apicomplexa*. Presence (filled dot) or absence (empty circle) of specific families of cyclin in each predicted proteome is shown. Dot area is proportional to number of putative proteins. The Group I cyclins in *Cryptosporidium* cannot be placed reliably into any specific families within the group (“Orphaned”). *The *Plasmodium berghei* predicted proteome (release 9.3; plasmodb.org/) contains no apparent orthologue of CYC1, as the likely gene encoding the protein on Chromosome 13 (downstream of PBANKA_132730, syntenic with *cyc1* in other *Plasmodium* species) is interrupted by a sequence gap.

To investigate the *Plasmodium* cyclin repertoire, we built a pan-cyclin hidden Markov model (HMM) of cyclins from a range of model species and used this to identify putative cyclins in a wide range of eukaryotes (see Methods). Our HMM showed good sensitivity, identifying full cyclin repertoires in diverse organisms not included in the seed alignment, and the resultant proteins sequences were aligned, trimmed to conserved regions and classified by constructing phylogenetic trees (Fig 1A and S1 Fig).

*Plasmodium* species encode only three identifiable cyclins–CYC1, CYC3 and CYC4. A *P*. *falciparum* gene (encoded by PF3D7_1227500) annotated as *Pf*CYC2 did not show a significant match to any of the cyclin-specific HMMs built during these analyses (even at extremely liberal thresholds), nor to the domains built by PFam. This protein was originally identified as a cyclin on the basis of a very limited similarity to a cyclin A from the sea star *Patiria (Asterina) pectinifera* (13% identity across alignable length) [35], but it lacks key alignable residues across most of the cyclin box and has no detectable cyclin-like function in biochemical assays [35]. These data strongly suggest that CYC2 is not a true cyclin and it is not included in the repertoire described here.

The *Plasmodium* cyclin repertoire is highly unusual in that it entirely lacks Group I, the largest group of cyclins. This group contains most canonical cyclin families that regulate specific cell cycle transitions with their CDK partners: Cyclin D-CDK4/6 for G1 progression, Cyclin E-CDK2 for the G1/S transition, Cyclin A-CDK2 for S phase progression and CyclinA/B-CDK1 for mitosis, although in fission yeast, all cell cycle transitions are driven by a single Cyclin B/CDK complex (CDC13/CDC2) [3,37]. In keeping with this key role and previous analyses [13], Group I cyclins were found in all non-apicomplexan species examined here, including the alveolate *Tetrahymena thermophila* [13]. However, none of the apicomplexan species examined contained Group I cyclins, except *Cryptosporidium*, which was found to encode three Group I cyclins of indeterminate family, suggesting that there has been a loss of Group I cyclins during the evolution of apicomplexan lineages (Fig 1B).

*Plasmodium* species encode only one cyclin from the P family (Group II) and two Group III cyclins from families H and L. The cyclin P family is not found in animals, but includes many plant cyclins and Pho80 in budding yeast, which link nutritional sensing to cell cycle progression. In contrast, both H and L families are associated with transcription: the CDK7/Cyclin H/MAT1 complex functions as a Cdk-activating kinase in cell cycle regulation [38] and as a modulator of the general transcription factor TFIIH [39,40]. Similarly, CDK11-Cyclin L complex in fission yeast regulates the formation of the Mediator complex, a coactivator of RNA polymerase II transcription [41].

### *Plasmodium* cyclins are transcribed at all developmental stages

**Fig 2:**
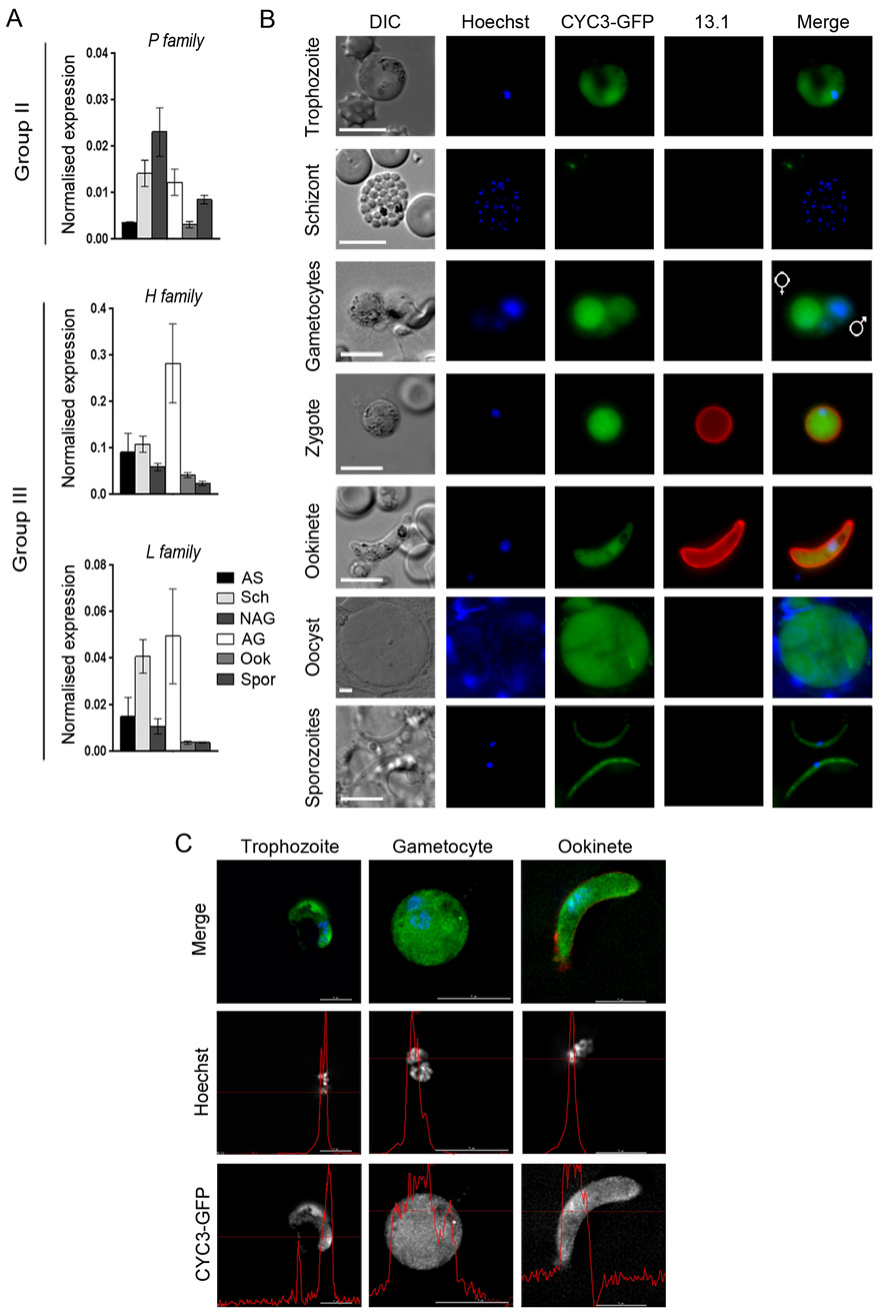
CYC3-GFP protein expression throughout most stages of the life cycle. (A) Transcription of *cyc1*, *cyc3* and *cyc4* as analysed by qRT-PCR, normalised against two endogenous control genes, *arginine-tRNA synthetase* and *hsp70*. Each bar is the mean of three biological replicates ± SEM. All asexual blood stages: AS; schizonts: Sch; non-activated gametocytes: NAG; activated gametocytes: AG; ookinete: Ook; 14 dpi oocysts/sporozoites: Spor. (B) Expression of CYC3-GFP in trophozoites, schizonts, gametocytes, zygotes, ookinetes, oocysts and sporozoites. 13.1, a cy3-conjugated antibody which recognises P28 on the surface of activated females, zygotes, and ookinetes was used with the sexual stages. Scale bar = 5 μm. (C) Deconvolved 2D projections of live trophozoite, gametocyte, and ookinete expressing CYC3-GFP (green), co-stained with Hoechst 33342 (blue) and cy3-conjugated anti-P28 antibody, 13.1 as a marker for the ookinete surface (red). Scale bar = 5 μm. Line profiles (red) in the black and white images indicate pixel intensity for that channel.

We used quantitative RT-PCR (qRT-PCR) to investigate the RNA levels of *cyc1*, *3* and *4* in six stages of the wild-type parasite life cycle. The transcription profiles showed expression of the cyclins throughout parasite development with the highest RNA levels for all three cyclins found in gametocytes (both non-activated and activated) and schizonts (particularly *cyc4*). The *cyc3* RNA level was highest in non-activated gametocytes, whereas for *cyc1* and *cyc4*, the levels were highest in activated gametocytes (Fig 2A). These results are similar to those obtained previously for *P*. *berghei* by RNA-seq analysis [42] and for *P*. *falciparum* using both RT-PCR [35] and RNA-seq [43].

### P-type CYC3-GFP is cytosolic and nuclear at most stages of the lifecycle

To investigate the localisation of CYC3, the only P-type cyclin in *Plasmodium*, throughout the *P*. *berghei* life cycle, we generated a C-terminal GFP fusion protein using single crossover recombination at the endogenous *cyc3* gene locus (PBANKA_123320; S2A Fig). Correct integration was confirmed by PCR, pulsed-field gel electrophoresis (PFGE) and Southern blot (S2B–S2D Fig). CYC3-GFP parasites were able to complete the full life cycle with no detectable phenotype observed from tagging with GFP including oocyst development at 14 days post infection (dpi) in mosquitoes (S2F and S2G Fig). Western blot with an anti-GFP antibody confirmed the expression of CYC3-GFP. A 54 kDa protein was detected in lysates from three stages of parasite development (schizonts, activated gametocytes and ookinetes) with the highest expression observed in activated gametocyte and ookinete stages compared with the 29 kDa GFP control extracted from a parasite line constitutively expressing GFP (GFPcon 507 cl1) [44] (S2E Fig).

Live imaging of parasites revealed CYC3-GFP presence throughout the parasite cell body with a predominantly cytosolic localisation at most of the key *Plasmodium* life cycle stages examined (trophozoite, male and female gametocyte, zygote, ookinete, oocyst and salivary gland sporozoite) (Fig 2B). However, we could not exclude the presence of CYC3-GFP also in the nucleus of trophozoites, gametocytes and ookinetes (Fig 2B). Therefore, for a more detailed analysis of the CYC3 localisation at trophozoite, schizont, gametocyte and ookinete stages, we used deconvolution fluorescence imaging (Fig 2C). Although no expression was detected in schizonts, two dimensional optical slices from 3D stacks showed that CYC3-GFP was uniformly present throughout both the cytoplasm and the nucleus in trophozoites, gametocytes and ookinetes (Fig 2C) and noticeably enriched in the nucleus of ookinetes (Fig 2C, line profile).

### CYC3 affects oocyst development, differentiation and sporozoite formation within the mosquito

To assess the function of CYC3 in the *Plasmodium* life cycle, we used a double crossover homologous recombination strategy to delete the *cyc3* gene (S3A Fig). Successful integration of the targeting construct at the *cyc3* locus was confirmed by diagnostic PCR across the junction of the expected integration site, as well as by Southern blot and PFGE (S3B–S3D Fig).

**Fig 3:**
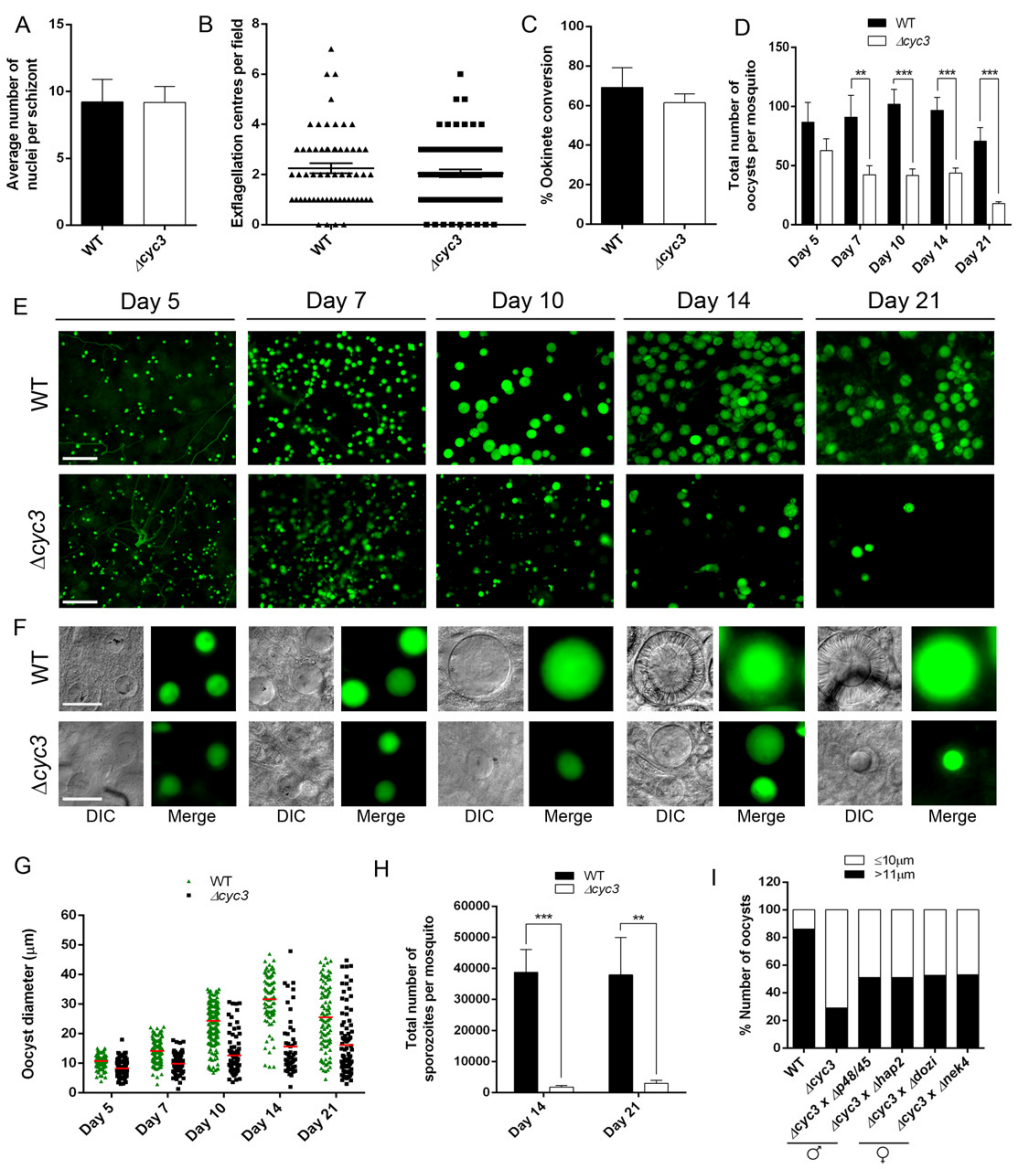
CYC3 is dispensable in asexual and sexual stages but important for oocyst development. (A) Average number of nuclei per schizont measured using Giemsa stained slides at 100x magnification. Bar is the mean ± SEM. n = 4 independent experiments. (B) Microgametogenesis of *∆cyc3* compared with WT measured as the number of exflagellation centres per field. Means ± SEM are shown. n = 4 independent experiments. (C) Ookinete conversion as a percentage in *∆cyc3* and WT lines. Ookinetes were identified using the marker 13.1 and defined as those cells that successfully differentiated into elongated ‘banana shaped’ ookinetes. Bar is the mean ± SEM. n = 5 independent experiments. (D) Total number of GFP-positive oocysts per infected mosquito, including normal and small oocysts, at 5, 7, 10, 14, 21 dpi for *∆cyc3* and WT lines. Bar is the mean ± SEM. n = 3 independent experiments (20 mosquitoes for each). Example of relative oocyst size and numbers at (E) 10x and (F) 63x magnification in *∆cyc3* and WT lines. Images show DIC and GFP at 5, 7, 10, 14 and 21 dpi. Scale bar = 100 μm for 10x and 20 μm for 63x. (G) Individual *∆cyc3* and WT oocyst diameters in μm at 5, 7, 10, 14 and 21 dpi. Horizontal line indicates the mean from 3 independent experiments (20 mosquitoes for each) of *∆cyc3* and WT. *p* <0.001 for all time points. (H) Total number of sporozoites per mosquito from 14 and 21 dpi midguts for *∆cyc3* and WT lines. Three independent experiments are described, n = 20 mosquitoes for each replicate. ** *p* ≤ 0.01, *** *p* ≤ 0.001. (I) Genetic complementation of *∆cyc3*. Mosquitoes were fed with a combination of WT, *∆cyc3* or *∆cyc3* with either male (∆*p48/45 and ∆hap2*) or female (*∆dozi and ∆nek4*) mutants. Shown is a representation of one experiment (20 mosquitoes per line).

Analysis of two independent *cyc3* deletion clones, *cyc3* cl1 and *cyc3* cl3 (hence forward called *Δcyc3*) showed no overt phenotype during blood stage asexual proliferation, microgamete exflagellation or ookinete conversion *in vitro* when compared with control parasites which constitutively express untagged GFP (WTGFPcon 507 cl1 line, henceforth known as WT) [44] (Fig 3A–3C).

To determine whether CYC3 is essential for parasite development in the mosquito vector, we fed female *Anopheles stephensi* mosquitoes with the *Δcyc3* mutant or WT parasites. There was no significant reduction in the number of *Δcyc3* oocysts compared to WT controls at 5 dpi, however a significant reduction was observed at 7 dpi, which became even more evident at 10, 14 and 21 dpi (Fig 3D and 3E).

Furthermore, oocysts at 14 and 21 dpi appeared substantially smaller in *Δcyc3* compared with WT lines (Fig 3F). To quantify this reduction in oocyst size, the diameter of WT and *Δcyc3* oocysts was measured at multiple time points during development in three independent mosquito infections (Fig 3G). Even at early stages during oocyst development (5 and 7 dpi), we already detected a difference in mean oocyst diameter between *Δcyc3* and WT parasites (8 μm and 11 μm for 5 dpi; 10 μm and 14 μm for 7 dpi, respectively) and by 10 dpi, the majority of *Δcyc3* oocysts were substantially smaller than WT (12 μm and 25 μm mean diameter, respectively) (Fig 3F and 3G). This difference in oocyst size increased dramatically at 14 and 21 dpi as WT oocysts reached maturity (mean diameter of 31 μm at 14 dpi compared to 15 μm for *Δcyc3* oocysts, Fig 3G). The majority of *Δcyc3* oocysts were smaller in size even at 5 dpi. After this, although a small number of the Δ*cyc3* oocysts continued to develop normally, the majority appeared to arrest and remained the same size until 21 dpi (Fig 3G).

In addition to the decrease in size, the number of *Δcyc3* sporozoites in infected mosquitoes was significantly reduced compared to WT in midguts at 14 and 21 dpi and in salivary glands at 21 dpi (Fig 3H, S4A and S4B Fig). However, when infected mosquitoes were allowed to feed on mice in bite-back experiments, we found that not only did transmission occur successfully but the pre-patent period for *Δcyc3* was the same as for WT parasites (S4A and S4B Fig). These data show that sporozoites produced from the few normal oocysts in the *Δcyc3* mutant are not affected in their efficacy of host infectivity.

Next we investigated whether the oocyst growth phenotype was related to a defect in ookinete structure, motility or DNA content. Ultrastructural analysis of ookinetes by transmission electron-microscopy revealed no morphological differences between *Δcyc3* and WT (S4C Fig). Similarly, both DNA content and the gliding motility of *Δcyc3* ookinetes were similar to WT (S4D and S4E Fig) suggesting that ookinetes are not affected by *cyc3* deletion.

Finally, we wanted to investigate whether *Plasmodium* CYC3 might function as a G1 or G2/M cyclin, which would explain the Δ*cyc3* phenotype during sporogony. To this end, we examined the ability of a codon-optimized version of the *P*. *berghei cyc3* gene to complement a triple *cln* (G1 cyclin) mutant of the budding yeast *Saccharomyces cerevisiae* or a temperature sensitive growth phenotype of a *cdc13-117* B-type cyclin mutant of the fission yeast *Schizosaccharomyces pombe* (G2/M transition), respectively (S5A and S5B Fig). In both cases, *P*. *berghei cyc3* failed to rescue yeast cyclin mutant strains, suggesting that *P*. *berghei* CYC3 does not function as a classical G1 cyclin or G2/M cyclin under these conditions.

### CYC3 functions in both male and female lineages

Since CYC3-GFP was expressed in gametocytes (Fig 2B), we examined whether the defect in oocyst formation was sex-specific. To do this we performed genetic crosses between *Δcyc3* parasites and lines deficient in either male (*Δp48/45* and *Δhap2*) or female (*Δdozi* and *Δnek4*) gametes [45–48]. As scored by an increase in normal size oocysts at 14 dpi, we found that all mutants could only partially rescue the phenotype of the *cyc3* knockout, which affected both male and female lines equally (Fig 3I). These results reveal that the functional *cyc3* is inherited through both male and female lines and its function is independent of sexual commitment at the gametocyte stage (Fig 3I).

### CYC3-GFP expression shows a temporal pattern during sporogony

**Fig 4:**
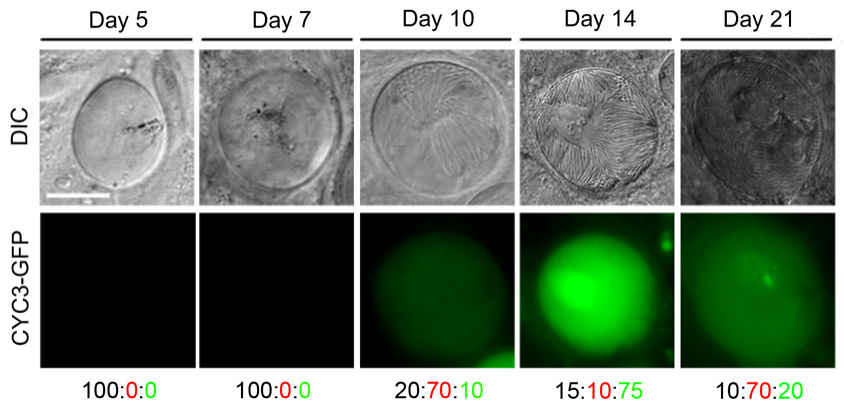
Expression of CYC3-GFP during sporogony in mosquitoes. Fluorescence microscopy of CYC3-GFP at different time points: 5, 7, 10, 14 and 21 dpi during development in the mosquito. Scale bar = 20 μm. Representative percentage of oocysts that either: do not express GFP (black number), have a low expression of GFP (red number) or have a high expression of GFP (green number).

As the Δ*cyc3* phenotype is observed during early oocyst development, we next examined CYC3-GFP expression during oocyst and sporozoite development at the same time points (5, 7, 10, 14 and 21 dpi) as described for the Δ*cyc3* phenotypic analysis within mosquitoes using fluorescence microscopy (Fig 4).

Fluorescent imaging showed no detectable CYC3-GFP expression in oocysts at 5 and 7 dpi (representative images in Fig 4). Expression of CYC3-GFP was first observed at low levels in the majority of oocysts at 10 dpi with the highest expression detected at 14 dpi. Oocysts that had formed fully mature sporozoites showed the highest protein expression. After 14 dpi, we observed a decrease in CYC3-GFP expression in oocysts up to day 21pi (Fig 4).

### Δ*cyc3* parasites show defects in cell growth and sporozoite budding, with abnormal membrane fusion and vacuolation

**Fig 5:**
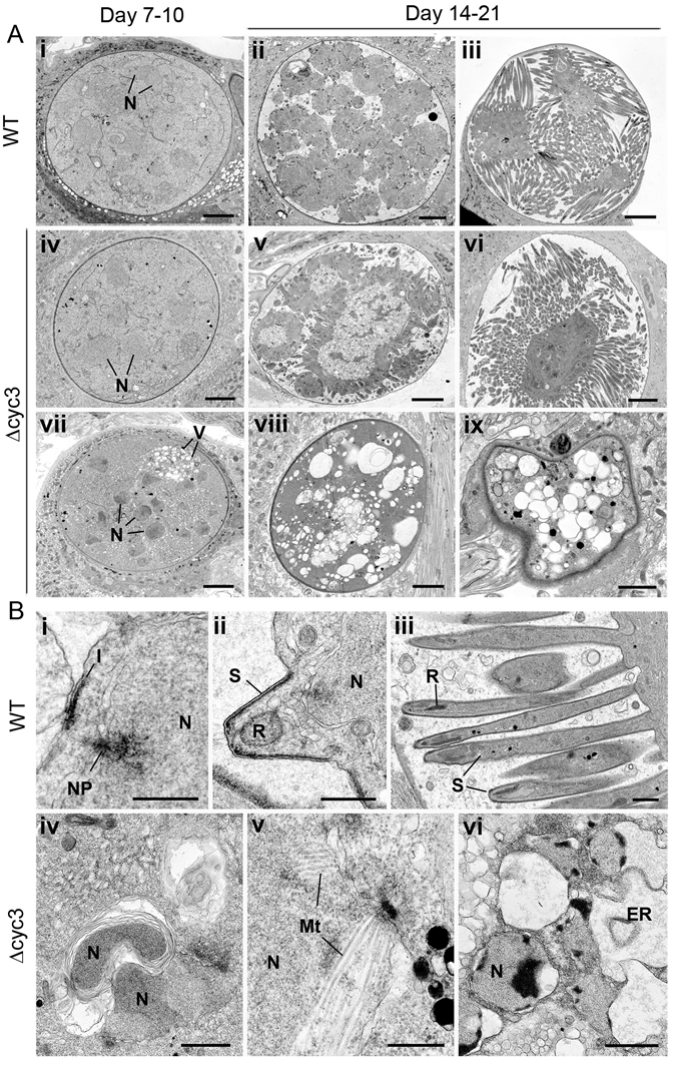
Ultrastructure analysis of oocyst development in *∆cyc3 mutant*. (A) Low power ultrastructural images of oocyst development at 7–10 days (i, iv, vii) and 14–21 days post-infection (dpi) showing normal sporulation of WT (ii, iii) and certain *Δcyc3* (v, vi) parasites while other *Δcyc3* mutants show evidence of cytoplasmic vacuolation (V) and degeneration (vii, viii, ix). N–nucleus. Bars represent 10 μm. (B) Details showing progressive stages in sporozoite formation in wild type parasites (i-iii) and various abnormal developmental stages of the *Δcyc3* parasite (iv-vi). Bars represent 1 μm. i. Initiation of sporozoite formation with formation of the inner membrane complex (I) beneath the plasmalemma and above a peripherally located nucleus (N) with nuclear pole (NP). ii. Early sporozoite (S) with rhoptry anlagen (R) budding from the surface of the sporoblast. N–nucleus. iii. Late stage in sporozoite formation showing the elongated sporozoites (S). R–rhoptry. iv. Detail of the sporoblast cytoplasm showing nuclei (N) enclosed by abnormal membrane whorls v. Part of a nucleus with extensive nuclear spindle microtubule (Mt) not seen in WT parasite. vi. Detail of a late stage in parasite degeneration showing apoptotic-like nuclei (N) and dilated endoplasmic reticulum (ER).

To define further the defect in oocyst growth during different developmental stages of sporogony, Δ*cyc3* and WT parasite-infected mosquito midguts at 7, 10, 14, and 21 dpi were examined by transmission electron microscopy. Marked differences were observed at the later time points in the ultrastructure of the majority of Δ*cyc3* compared to WT oocysts, although some Δ*cyc3* oocysts appeared similar to WT (Fig 5). At every time point there were significantly fewer oocysts in the guts of mosquitoes infected with Δ*cyc3* compared to the WT parasites (5 oocysts/gut Δ*cyc3* compared to 60 oocyst/gut WT at 7 dpi).

At 7 and 10 dpi the oocysts of both the WT and Δ*cyc3* had similar structural appearance being spherical with the cytoplasm completely filling the cyst (Fig 5Ai, 5Aiv and 5Avii). The cytoplasm contains numerous nuclear profiles and homogenous appearing cytoplasm containing mitochondria and apicoplasts. However, a proportion (approximately 10%) of the Δ*cyc3* oocysts also showed centrally located nuclei and exhibited some vacuolation of the cytoplasm (Fig 5Avii). By 14 dpi, the majority (>98%) of WT oocysts exhibited various stages of sporozoite formation (Fig 5Aii, 5Aiii and 5Bi–5Biii) or oocysts with mature sporozoites (Fig 5Aiii). In contrast, there were many fewer Δ*cyc3* oocysts (10 Δ*cyc3* compared to 60 WT oocysts) and only a proportion (<40%) of these showed similar features of sporulation (Fig 5Av and 5Avi). Many (>60%) of the Δ*cyc3* oocysts showed no evidence of sporulation (Fig 5Aviii and 5Aix). These oocysts showed no retraction of the plasmalemma to form the sporoblasts and there was no evidence of the initiation of sporozoite formation (Fig 5Aviii and 5Aix). However, cytoplasmic changes were observed including abnormal membrane reduplication (Fig 5Biv) and nuclei containing large numbers of microtubules, suggesting mitosis and cytokinesis were affected (Fig 5Bv). There was evidence of nuclei with apoptotic-like chromatin changes and dilated endoplasmic reticulum (Fig 5Bvi). There appeared to be continued cytoplasmic vacuolation consistent with progressive cell death (Fig 5Aviii, 5Aix and 5Bvi). These results are consistent with the idea that DNA synthesis, endomitosis and cytokinesis are severely defective in most but not all oocysts. Moreover, structural abnormalities in some Δ*cyc3* oocysts suggest that they are incapable of forming viable sporozoites.

### Transcriptome analysis of *Δcyc3* parasites reveals modulated expression of genes involved in signalling, ookinete invasion, and oocyst development

**Fig 6:**
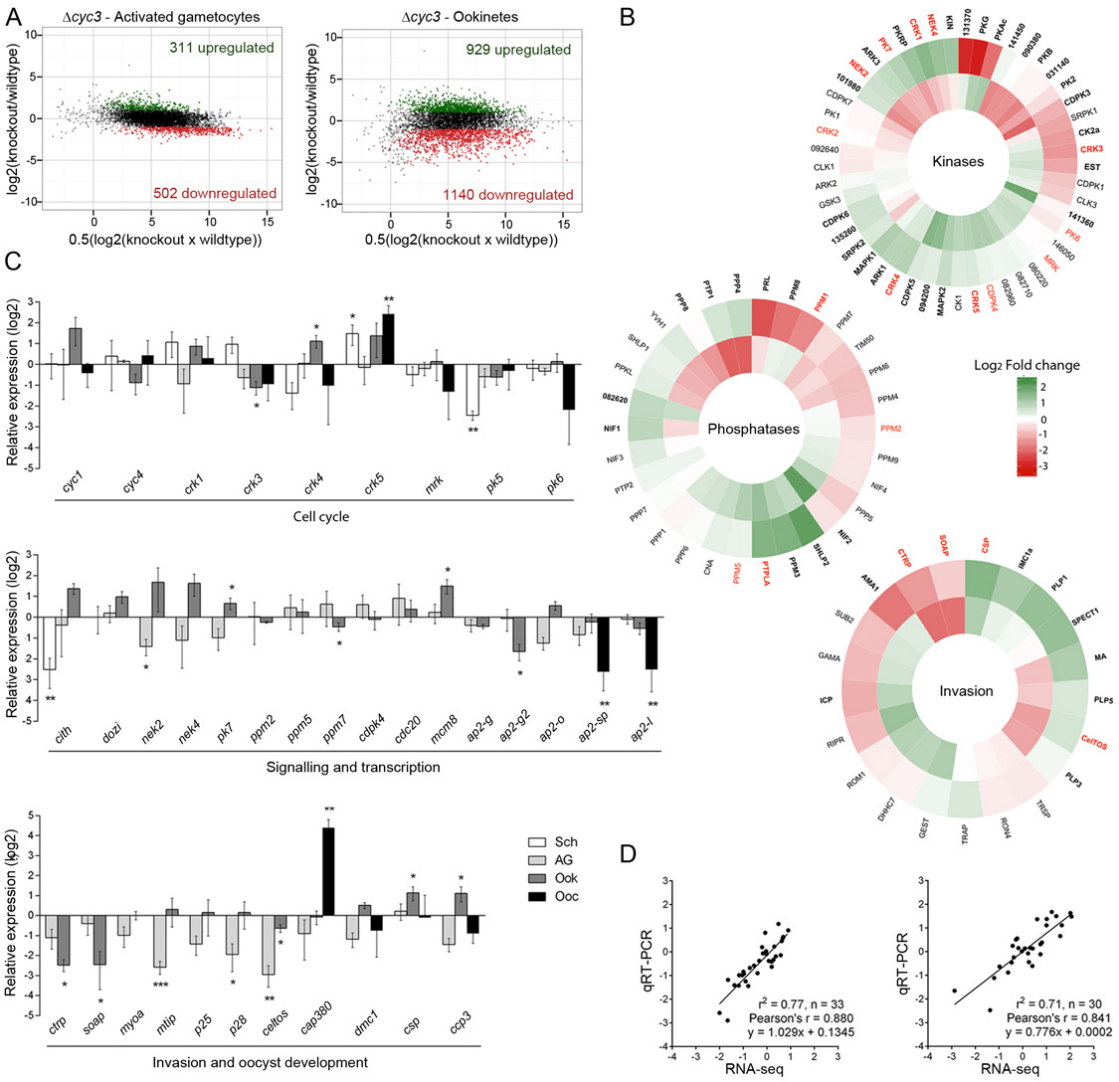
Transcript analysis of genes involved in parasite development. (A) Ratio-Intensity scatter plots for ∆*cyc3* activated gametocytes and ookinetes. The y-axis shows the log_2_ fold change between wild-type and mutant and the x-axis shows the average of normalised FPKM (See also S2 Table). Significantly up-regulated genes are highlighted in green while down-regulated genes are highlighted in red. (B) Heatmaps for invasion, kinase and phosphatase gene clusters based on their log_2_ fold change in *Δcyc3* activated gametocytes (inner track) and *Δcyc3* ookinetes (outer track) relative to WT. Functional groups were inferred from annotations available in GeneDB (http://www.genedb.org/). Genes that were found significantly misregulated are shown in bold and those validated by qRT-PCR are shown in red. Full gene list and functional clusters are shown in S3 Table. (C) Log_2_ fold transcript change in Δ*cyc3* at different life-cycle stages of cell cycle, signalling and transcription genes, and invasion and oocyst development genes, studied in ∆*cyc3* (compared against WT) using qRT-PCR. Data were normalised against an endogenous control gene, *hsp70* (PBANKA_081890). Each bar is the mean of relative expression in comparison to WT from three biological replicates ± SEM. Sch: schizonts; AG: activated gametocytes; Ook: ookinetes; Spor: 14 dpi oocysts/sporozoites. * *p* ≤0.05; ** *p* ≤0.01, *** *p* ≤0.001. (D) Comparison of qRT-PCR and RNA-seq data (Cuffdiff2 analysis) using Log_2_ values for activated gametocyte and ookinete samples.

The marked changes in *Δcyc3* oocyst morphology and growth led us to analyse the regulation of mRNA in *Δcyc3* parasites compared with WT. We first used strand-specific RNA sequencing (RNA-seq) to investigate the global transcript levels in *Δcyc3* and WT activated gametocytes and ookinetes. The deletion of *cyc3* was confirmed by RNAseq, with no reads mapping to the region of the gene targeted for disruption (S6A Fig). Generally, most transcript levels were very similar in activated gametocytes, with strong linkage between levels in *Δcyc3* and WT lines. However, the ookinete transcriptome was greatly altered by loss of *cyc3*, with many genes down-regulated and a smaller number up-regulated. In total, 813 and 2,069 genes showed modulated expression in activated gametocytes and ookinetes, respectively (p <0.05 and fold change >2; 702 and 1,891 genes in activated gametocytes and ookinetes, respectively, with p <0.01 and fold change >2) (Fig 6A, S6B Fig and S2 Table), including those with roles in reversible phosphorylation, transcription, cell signalling and inner membrane complex function (Fig 6B, S6C Fig and S3 Table). We identified several functional clusters that were significantly differentially expressed in *Δcyc3* (Fig 6B, S6C Fig and S3 Table) which may be collectively responsible for the observed phenotype. Global Gene Ontology (GO) analysis showed enrichment of genes associated with GO terms ‘kinase activity’, ‘protein phosphorylation’ and’ inner membrane complex’ (S6D Fig).

To validate our RNA-seq data, we performed qRT-PCR analysis of specific sets of genes known to have roles in cell cycle, signalling and transcription, or invasion and oocyst development (Fig 6C and S2 Table). These data showed very good correlation with the RNA-seq results (Fig 6D) validating the results for activated gametocytes and ookinetes. In addition, qRT-PCR data were collected for schizonts and/or day 14 oocysts (Fig 6C); these data showed that most of the changes in transcript levels for these sets occur at the ookinete stage, with some additional effects in oocysts.

Importantly, both RNA-seq and qRT-PCR analyses showed that neither of the other two *Plasmodium* cyclin genes were differentially expressed in response to deletion of *cyc3*, suggesting that there is no compensation provided by up-regulation of other cyclins. In contrast, several of the *cdks* were differentially expressed at different stages (Fig 6C): of particular interest was *crk1* and *crk5*, which were significantly up-regulated in ookinetes and are known to have roles in gene transcription [49] and cell cycle [50], respectively.

Recently, it has been shown that a family of transcription factors (apiAP2) is responsible for gametocyte commitment, as well as ookinete and sporozoite development [36,51–55]. This family is also a feature of chromerid genomes and their evolutionary history suggests they have mediated evolutionary changes during lineage differentiation [56]. Our analysis showed that several apiAP2 family members were affected by *cyc3* deletion, notably a significant down-regulation of *ap2-sp* and *ap2-l* in *Δcyc3* ookinetes and oocysts, consistent with a reduction in normal oocyst formation (Fig 6C, S6C Fig and S2 Table). Due to the important role of reversible phosphorylation in cell cycle progression in other systems [3], we next looked at the expression of protein kinases, phosphatases and cell cycle proteins involved in parasite development, especially those that either display a similar phenotype to that of *Δcyc3* when deleted (*pk7*, *ppm5*) or are involved in sexual development (*nek4*). The expression of genes involved in male sexual development (*cdpk4* and *cdc20)* [29,57,58] was not significantly affected. Furthermore, the transcription of *ppm7*, *ppm5* and *pk7*, which have similar protein localisation and/or similar phenotypes in gene knock-out lines [30,31], were also unaffected (Fig 6C). However, two genes important for zygote differentiation (*nek2* and *nek4)* [47,59] were significantly mis-regulated in activated gametocytes and ookinetes. Based on the phenotype observed in *Δcyc3* parasites and the fact that several known invasion genes were significantly affected, as detected in the RNA-seq analysis, we next focused on genes involved in ookinete invasion and oocyst development. Interestingly, there was a marked down-regulation of genes required for ookinete motility and invasion (Fig 6C), such as *mtip*, *ctrp* and *soap* [60,61], despite the fact that ookinete development and motility appeared to be unaffected (S4 Fig). Genes known to be required during oocyst development, such as *cap380*, coding for a capsule protein necessary for healthy oocyst formation and subsequent transmission [62], and *csp*, important for infectivity of the host [63], were mis-regulated in multiple stages (Fig 6C). These data suggest that the oocyst development phenotype in Δ*cyc3* may be a downstream consequence of the mis-regulation of these genes, well before sporogony commences.

Overall, deletion of *cyc3* caused the modulated expression of a number of genes related to the observed phenotype, especially in ookinetes, including transcription factors, putative cyclin binding partners, and genes for signalling and subsequent oocyst development.

## Discussion

The molecular mechanisms controlling the cell cycle and mitosis are regulated by key molecules including CDKs and cyclins. Cyclins are key regulators of CDKs and are synthesised and degraded at specific phases of the cell cycle [5,64]. Although cdk/cyclins have been well studied in many eukaryotic model systems, their role in the unicellular parasite *Plasmodium* is not well understood. Here, we show that despite its complex and atypical pattern of cell cycle and division, *Plasmodium* only has three cyclin genes, a very small complement compared to other organisms including human (30 annotated genes in the Refseq dataset), yeast (22 genes) and *Drosophila* (12 genes). *Plasmodium* has no Group I cyclins but has homologues belonging to Group II (CYC3) and Group III (CYC1 and CYC4). CYC3 is a P-type cyclin, a family which contains many cyclins of plants (CYCPs) [17]. It also shows similarity to PHO80 cyclin from *Saccharomyces cerevisiae* [20] and a G1 cyclin present in kinetoplastids [65,66]. Both Pho80 and plant CYCPs appear to link cell cycle regulation to nutritional status: in yeast, Pho80 and other P-like cyclins associate with CDK Pho85 to phosphorylate a variety of substrates involved in phosphate, glycogen and carbon source metabolism (reviewed in [16]), whereas CYCP2;1 in *Arabidopsis* is required for G2/M transition in meristem activation in a nutrition-responsive manner [18]. Trypanosomes encode at least three P-type cyclins, CYC2, 4, and 7, with CYC10 and 11 being likely divergent members of the family (“Pho80-like” cyclin *Tb*CYC5 [66] is a cyclin Y, see S1 Fig). In contrast to CYCP, *Trypanosoma brucei* CYC2 is required for progression from G1 phase in the insect stage of the parasite [67], which would put its cell cycle activity in a position more akin to the Pho80-like cyclins (Pcls) in yeast [16]. In keeping with this, *Tb*CYC2 is able to rescue a yeast CLN mutant [19], in which 3 Group I G1 cyclins, *CLN1*, *2* and *3*, were inactivated [68].

There are only three clearly identifiable CDKs (*Pf*PK5, *Pf*crk1 and *Pf*crk3) in the *Plasmodium* genome [31,69]. The lack of cyclin D in *Plasmodium* here mirrors the absence of CDK4/6 orthologues previously reported [49], and *Pf*crk1 is a CDK10/11-related protein [69], which matches well with the presence of cyclin L family members identified here. Moreover, a fourth protein, *Pf*mrk, was initially described as a CDK7 homologue and was shown to be stimulated by binding to human cyclin H *in vitro* [70], but subsequent work showed that its affinities are less clear [69] and functional work suggests it might be a RNA polymerase II carboxyl-terminal domain kinase rather than a CDK [71]. There is no homologue of Pho85 identified in *Plasmodium* and the essential kinase *Pf*PK5 is a relative of CDK1/2, which would normally be activated by a Group I cyclin in animals or fungi.

Transcription of *P*. *berghei* cyclins varies during different phases of the life cycle with the highest transcription seen in gametocytes and schizonts [35]. Our localisation studies of CYC3-GFP by fluorescence and deconvolution microscopy showed both cytoplasmic and nuclear localisation, which is expected given the localisation and role of most cyclins in model systems [10]. The subcellular location of P-type cyclins (for example, CYCP2;1) in plants is mainly nuclear [18], however, *Tb*CYC2 is also expressed in the cytosol of procyclic forms and controls posterior morphogenesis of the parasite during the G1/S and G2/M transition [19,65,66,72].

A recent study on the AP2-O transcription factor in *Plasmodium* showed that *cyc3* is a target of AP2-O and used deletion of *cyc3* as a validation tool to show that it had a role in ookinete to oocyst development [36]. Our gene deletion mutant is consistent with this analysis in terms of number and size of oocysts, but further dissects in depth the function of *cyc3* throughout the lifecycle using transcriptome analysis and high resolution microscopy. Maturation of most of the *∆cyc3* oocysts, based on their abnormal shape and size, was impaired in infected mosquito guts, and detailed studies revealed that this defect in oocyst growth and differentiation began during early sporogony. *Plasmodium* oocysts contain a highly lobed syncytial nucleus that divides at the time of sporozoite budding into a number of lobes, which undergo subsequent mitotic division resembling endomitosis [23,25]. The *∆cyc3* phenotype suggests that although initial oocyst formation occurs, in many of the oocysts the division of nuclear lobes with oocyst growth is drastically affected and further budding of sporozoites from these lobes does not occur. This defect leads to abnormalities in membrane formation, vacuolation and subsequent cell death during the later stages of sporogony. The characteristics of *∆cyc3* oocyst development suggest that maturation and differentiation are arrested, with cells unable to progress further to form additional lobes and start sporozoite budding and mitotic division.

A number of gene deletion mutants have been described in *Plasmodium* that affect oocyst maturation for example; *Pb*CAP380, affecting oocyst capsule formation [62]; *Pb*GEX and *Pb*DMC1, affecting oocyst size and sporozoite formation [73,74]; *Pb*LAPs and *Pb*CDLK, affecting sporogony but not the size of the oocyst [31,75,76]; *Pb*MISFIT, causing small sized oocysts [77]; and G actin sequestering protein (C-CAP) affecting oocyst development and showing similar features to *Δcyc3* during early oocyst development [78]. However, the phenotype of *Δcyc3* lines differs from that of all these mutants due to the fact that some normal oocysts are produced, which are able to form invasive salivary gland sporozoites and initiate liver stage infection. Moreover, none of the other mutated genes was implicated in cell cycle control or cell division. The presence of a small number of normal oocysts/sporozoites in the Δ*cyc3* mutant does suggest that CYC3 has a subtle role in oocyst/sporozoite development. The reduction in sporozoite number (in addition to the reduction in oocyst number) is drastic at 14 and 21 dpi compared to the WT parasites and there is clear expression of CYC3-GFP in 14 dpi oocysts, both indicative of a role in sporozoite development. This might suggest that there are unknown regulatory mechanisms involved in the control of the oocyst development that can by-pass a default CYC3-dependent pathway to some degree in these parasites during early oocyst development and sporogony.

Specific protein kinase and phosphatase mutants (*Pb*PK7 and *Pb*PPM5) show some resemblance to the Δ*cyc3* phenotype, with a reduced number of oocysts, which were abnormally small and did not complete sporogony before arresting [30,31]. However, only minor changes in the transcript level of protein kinase (*pk7)* or phosphatase *(ppm5)* were detected. Conversely, *cyc3* transcripts are down-regulated in *Δppm5* parasites [30] suggesting that PPM5 acts upstream of CYC3.

Gene deletion revealed that CYC3 is dispensable during asexual multiplication in erythrocytes and in the liver (erythrocytic and exo-erythrocytic schizogony) and sexual development during male gametogenesis. However, CYC3 clearly modulates sporogony via endomitotic multiplication during oocyst development in the mosquito. Oocysts are the only replicative extracellular stage during the parasite life cycle, and therefore it is possible that CYC3 modulates this extracellular replication in response to metabolic sensing within the mosquito. For example, *Arabidopsis* CYCP2;1 is transcribed in response to sugar signals by a specific transcriptional factor, allowing cell cycle progression [18]. CYC3 may have a direct or indirect role in regulation of transcription in the ookinete, of specific genes involved in oocyst growth (defined as a G1 phase) and sporogony (defined as S/mitotic phases), and that could explain why we see expression of CYC3-GFP in ookinetes and 10 to 14 dpi oocysts. Rescue experiments in yeast suggest that *Plasmodium* CYC3 does not behave as a classical G1 or G2/M cyclin, or at least cannot substitute for the function of yeast cyclins. Nevertheless the peak in expression of CYC3-GFP around 10 to 14 dpi of oocyst development, just before sporogony, and the defect in sporozoite production in *Δcyc3* mutants, are both consistent with a cyclin-B-like role in cell cycle progression and a role in G1/S progression.

Global transcript analysis of the *Δcyc3* mutant suggested that neither *cyc1* nor *cyc4* compensates for the *cyc3* deletion. Analysis of all the CDKs showed that several, including *crk1*, with a role in transcription [49], were mis-regulated at several stages in *Δcyc3* parasites; however it is unlikely that transcription of a putative CDK partner would be controlled by CYC3. It has been reported previously that *Pf*CYC3 binds and activates the CDK1 homologue, *Pf*PK5, *in vitro* [35], and a similar result was reported for the homologues from *Eimeria tenella* [79], although we saw no significant mis-regulation of *pk5* in *Δcyc3* parasites and no study has shown a *Plasmodium* cyclin-CDK interaction *in vivo*. Other cell cycle genes were affected including predicted members of the mini chromosome maintenance (MCM) family. While no MCM has been functionally characterised in *Plasmodium*, the MCM family has been bioinformatically well classified in *Apicomplexa* [80,81] and MCMs are known to be important for G1/S phase progression and initiation of DNA replication [82].

Misregulation of genes involved in ookinete invasion and structure (such as *mtip*, *ctrp* and *soap*) had no observable effect on ookinete motility or oocyst formation at 5 dpi and we observed no detectable phenotype with electron microscopy, however, a subtle delay in oocyst initiation (due to ookinete invasion or motility) may be enough to initiate deleterious consequences downstream during oocyst development. In addition to this, we cannot rule out the possibility that CYC3 is indirectly involved in nutrient/environmental sensing in the mosquito gut, a known function of cyclins in other systems [18]. The mis-regulation of these, as yet undefined, genes may be responsible for the mixed population of small, defective oocysts versus normal, healthy oocysts. Thus, the absence of *cyc3* may make these parasites more sensitive to environmental stimuli such as nutrient abundance, or the impact of developmental mis-timing (via mis-regulation of invasion genes).

Oocyst growth may further deteriorate following the mis-regulation of genes required for oocyst development (such as *cap380*). Transcription analysis also showed that several members of the apiAP2 transcription factor family, which have been shown to regulate various stages of development, were affected in *∆cyc3* oocysts. This is perhaps unsurprising considering that normal regulation of these genes is required for successful oocyst development [52,53,55]. Global transcriptomics is a useful tool for the identification of possible dysregulation and compensatory mechanisms in *Δcyc3* parasites however the measurement of mRNA levels is not necessarily indicative of the corresponding protein levels. Future proteomic work may provide more information on protein-protein interactions betweenCYC3 and putative CDK partners or on the effects of *cyc3* deletion on global (or cell cycle specific) protein levels in the *Δcyc3* mutant.

In conclusion, this is the first study to classify phylogenetically the cyclins in *Plasmodium* and uncover, in depth, an important function for CYC3, a novel P-type cyclin. We describe a key role for this cyclin during the early stages of ookinete to oocyst development, specifically the G1/S phase, which subsequently affects differentiation and sporogony, suggesting it is a modulator of transcription and oocyst endomitotic development in *Plasmodium*.

## Materials and Methods

### Ethics statement

All animal work at Nottingham has passed an ethical review process and was approved by the United Kingdom Home Office. Work was carried out in accordance with the United Kingdom ‘Animals (Scientific Procedures) Act 1986’ and in compliance with ‘European Directive 86/609/EEC’ for the protection of animals used for experimental purposes under UK Home Office Project Licenses 40/3344 and 30/3248. Sodium pentabarbitol was used for terminal anaesthesia and a combination of ketamine followed by antisedan was used for general anaesthesia. All efforts were made to minimise animal suffering.

### Animals

Six-to-eight week old female Tuck-Ordinary (TO) (Harlan) outbred mice were used for all experiments.

### Bioinformatic analysis of cyclins

To identify cyclin-like proteins, a pan-cyclin hidden Markov model was used to perform a similarity search using HMMER3 [83]. Briefly, all annotated cyclins were taken from the predicted proteomes of human, *Arabidopsis thaliana*, *Caenorhabditis elegans*, *Saccharomyces cerevisiae*, *Schizosaccharomyces pombe*, and *Toxoplasma gondii*. These cyclins were aligned using MAFFTv6.925b [84] with the L-INS-i strategy [85], trimmed to conserved regions and used to create a HMM, which was used to search the predicted proteomes of 12 apicomplexan parasites (EuPathDB; http://eupathdb.org; see Fig 1C) as well as 20 other eukaryotes from diverse groups. The conserved cyclin domains were excised from these sequences based on HMM hits with e-values ≤ 10^−18^, realigned and used to create a refined HMM, which was then used to re-search the apicomplexan proteomes at a threshold of e-value ≤ 10^−12^. Alignments of cyclins were trimmed to conserved regions and used to infer maximum likelihood phylogenies as implemented by the program PhyML3.0 [86] using the LG substitution matrix with a gamma-distributed variation in substitution rate approximated to 4 discrete categories (shape parameter estimated from the data). Trees shown are a majority-rule consensus of 100 bootstrap replicates for each alignment.

### Generation of transgenic parasites

For GFP-tagging by single homologous recombination [30], a 986 bp region of *cyc3* starting 332 bp upstream of the ATG start codon and omitting the stop codon was amplified using primers T0891 and T0892. This was inserted upstream of the *gfp* sequence in the p277 vector using KpnI and ApaI restriction sites. The p277 vector contains the human *dhfr* cassette, conveying resistance to pyrimethamine. Before transfection, the sequence was linearised using HindIII,

The gene targeting vector for ∆*cyc3* lines was constructed using the pBS-DHFR plasmid, which contains polylinker sites flanking a *T*. *gondii dhfr/ts* expression cassette conveying resistance to pyrimethamine, as described previously [31]. PCR primers N0451 and N0452 were used to generate a 411 bp fragment of 5′ upstream sequence of *cyc3* from genomic DNA, which was inserted into ApaI and HindIII restriction sites upstream of the *dhfr/ts* cassette of pBS-DHFR. A 663 bp fragment generated with primers N0453 and N0454 from the 3′ flanking region of *cyc3* was then inserted downstream of the *dhfr/ts* cassette using EcoRI and XbaI restriction sites. The linear targeting sequence was released using ApaI/XbaI. All of the oligonucleotides used to make these constructs can be found in S1 Table.

*P*. *berghei* ANKA line 2.34 (for GFP-tagging) or ANKA line 507cl1 (for gene deletion [44]) were then transfected by electroporation [44]. Briefly, electroporated parasites were mixed immediately with 100 μl of reticulocyte-rich blood from a phenylhydrazine (6mg/ml, Sigma) treated, naïve mouse, incubated at 37°C for 20 min and then injected intraperitoneally. From day 1 post infection pyrimethamine (70 μg/ml, Sigma) was supplied in the drinking water for four days. Mice were monitored for 15 days and drug selection was repeated after passage to a second mouse. Resistant parasites were then used for cloning by limiting dilution and subsequent genotyping.

### Genotypic analysis of mutants

For the C-terminal fusion GFP-tagged parasites, a diagnostic PCR reaction was used as illustrated in S2 Fig. Primer 1 (INT T89) and Primer 2 (ol492) were used to determine correct integration of the *gfp* sequence at the targeted locus (S1 Table). After confirmation of correct integration, genomic DNA from wild type and transgenic parasites was digested with BsmI and the fragments were separated on a 0.8% agarose gel, blotted onto a nylon membrane, and probed with a PCR fragment homologous to the *P*. *berghei* genomic *cyc3* sequence cloned in the p277 vector, using the Amersham ECL Direct Nucleic Acid Labelling and Detection kit (GE Healthcare). Parasites were also visualised on a Zeiss AxioImager M2 (Carl Zeiss, Inc) microscope fitted with an AxioCam ICc1 digital camera (Carl Zeiss, Inc) and analysed by Western blot to confirm GFP expression and the correct protein size.

For the gene knockout parasites, two diagnostic PCR reactions were used as shown in S3 Fig. Primer 1 (INT N45) and primer 2 (ol248) were used to determine successful integration of the selectable marker at the targeted locus. Primers 3 (N45 KO1) and 4 (N45 KO2) were used to verify deletion of the gene. After confirmation of integration, genomic DNA from wild type and mutant parasites was digested with HindIII and the fragments were separated on a 0.8% agarose gel, blotted onto a nylon membrane (GE Healthcare), and probed with a PCR fragment made with primers N0453 and N0454 which is homologous to the *P*. *berghei* genomic DNA just outside of the targeted region. All of the oligonucleotides used to genetically confirm these mutant parasite lines can be found in S1 Table.

Chromosomes of wild type, gene knockout and GFP-tagged parasites were separated by pulsed field gel electrophoresis (PFGE) on a CHEF DR III (BioRad) using a linear ramp of 60–500 s for 72 h at 4 V/cm. Gels were blotted and hybridized with a probe recognizing both the resistance cassette in the targeting vector and, more weakly, the 3′UTR of the *P*. *berghei dhfr/ts* locus on chromosome 7.

### Phenotypic analysis

To record the nuclei number per schizont, merozoites in late schizonts were counted 18–24 hours after schizont cultures were made. Exflagellation was examined on day 4 to 5 post-infection. Ten μl of gametocyte-infected blood was obtained from the tail with a heparinised pipette tip and mixed immediately with 40 μl of ookinete culture medium (RPMI1640 containing 25 mM HEPES, 20% fetal bovine serum, 10 mM sodium bicarbonate, 50 μM xanthurenic acid at pH 7.6). The mixture was placed under a Vaseline-coated cover slip and 15 min later exflagellation centres were counted by phase contrast microscopy in 12–15 fields of view using a 40× objective and 10× ocular lens. Ookinete formation was assessed the next day. Ten μl of infected tail blood was obtained as above, mixed immediately with 40 μl ookinete culture medium, and incubated for 2 hours at 20°C to allow completion of gametogenesis and fertilisation. Each culture was then diluted with 0.45 ml of ookinete medium and incubated at 20°C for a further 21–24 hours to allow ookinete differentiation. Cultures were pelleted for 2 min at 5000 rpm and then incubated with 50 μl of ookinete medium containing Hoechst 33342 DNA dye to a final concentration of 5 μg/ml and a Cy3-conjugated mouse monoclonal antibody 13.1 [58] recognizing the P28 protein on the surface of ookinetes and any undifferentiated macrogametes or zygotes. P28-positive cells were counted with a Zeiss AxioImager M2 microscope (Carl Zeiss, Inc) fitted with an AxioCam ICc1 digital camera. Ookinete conversion was expressed as the percentage of P28 positive parasites that had differentiated into ookinetes [45].

For mosquito transmission experiments 20–50 *Anopheles stephensi* SD500 female mosquitoes were allowed to feed for 20 min on anaesthetised infected mice whose asexual parasitaemia had reached ~12–15% and were carrying comparable numbers of gametocytes as determined by Giemsa stained blood films. Days 5, 7, 10, 14, and 21 days post-infection (dpi) 20 mosquitoes were dissected and oocysts on their midguts counted. Oocyst formation was examined following Hoechst 33342 staining in PBS for 10–15 min and guts were mounted under Vaseline-rimmed cover slips. Counting and images were recorded using 10x and 63x oil immersion objectives on a Zeiss AxioImager M2 microscope fitted with an AxioCam ICc1 digital camera. At 14 and 21 dpi, the same mosquito midguts used to record the oocyst number were homogenised in a loosely fitting homogeniser to release sporozoites, which were then quantified using a haemocytometer. Only for 21 dpi mosquitoes, salivary glands were dissected and homogenised in a loosely fitting homogeniser to release sporozoites, which were then quantified using a haemocytometer. Due to day-to-day variations in transmission levels, all data were normalised to a matching number of wild type controls analysed on the same day. Mosquitoes infected with wild type or *Δcyc3* parasites were used to perform bite back experiments with a TO mouse each in three independent experiments. Blood stage parasitaemia was measured for wild-type and *Δcyc3* by Giemsa staining at 4 dpi.

Oocyst diameter was measured with the AxioVision Imager software from images of 50–60 oocysts, in triplicate for 5, 7, 10, 14 and 21 dpi using a 63x oil immersion objective on a Zeiss AxioImager M2 microscope fitted with an AxioCam ICc1 digital camera.

For genetic complementation, we used either male (*∆p48/45 and ∆hap2*) or female (*∆dozi and ∆nek4*) parasites using a method described previously [30]. Briefly, mice were infected with combinations of the different parasite lines mentioned above and subsequently fed to 3–6 day old mosquitoes. These were dissected 12–14 dpi and the diameter of oocysts was determined as mentioned above. Statistical analyses were performed using GraphPad Prism (GraphPad Software). For comparison between *Δcyc3* and WT, an unpaired Student’s *t*-test was used.

For the fluorescence pictures of CYC3-GFP oocysts, mosquito midguts have been dissected at 5, 7, 10, 14 and 21 dpi and images were recorded using 63x oil immersion objectives on a Zeiss AxioImager M2 microscope fitted with an AxioCam ICc1 digital camera.

### Western blotting

Schizont, gametocyte and ookinete samples were isolated as described below. WT-GFP or CYC3-GFP samples were then purified using a GFP-Trap kit to immunoprecipitate GFP-fusion protein (Chromotek). After the addition of Laemmli sample buffer, the samples were boiled and an equal concentration of total protein was loaded on a 4–12% SDS-polyacrylamide gel. Samples were subsequently transferred to nitrocellulose membranes (Amersham Biosciences) and immunoblotting performed using the Western Breeze Chemiluminescent Anti-Rabbit kit (Invitrogen) and anti-GFP polyclonal antibody (Invitrogen) at a concentration of 1:1250, according to the manufacturer's instructions.

### Electron microscopy

Ookinete samples (described below) and mosquito midguts at 7, 10, 14 and 21 dpi were fixed in 4% glutaraldehyde in 0.1 M phosphate buffer and processed for routine electron microscopy as previously described [87]. Briefly, samples were post fixed in osmium tetroxide, treated *en bloc* with uranyl acetate, dehydrated and embedded in Spurr's epoxy resin. Thin sections were stained with uranyl acetate and lead citrate prior to examination in a JEOL1200EX electron microscope (Jeol UK Ltd).

### Purification of schizonts, gametocytes, ookinetes and oocysts

Purification of gametocytes was achieved using a protocol modified from [88]. Mice were treated by intra-peritoneal injection of 0.1 ml of phenylhydrazine (6 mg/ml,Sigma) in PBS to encourage reticulocyte formation four days prior to infection with parasites. Four days after parasites injection in mice, mice were treated with sulfadiazine (Sigma) at 20 mg/L in their drinking water for two days to eliminate asexual blood stage parasites. On day six post-injection (p.i), mice were bled by cardiac puncture into heparin and gametocytes separated from uninfected erythrocytes on a 48% NycoDenz gradient (27.6% w/v NycoDenz in 5 mM Tris-HCl, pH 7.20, 3 mM KCl, 0.3 mM EDTA) in coelenterazine loading buffer (CLB), containing PBS, 20 mM HEPES, 20 mM Glucose, 4 mM sodium bicarbonate, 1 mM EGTA, 0.1% w/v bovine serum albumin, pH 7.25. Gametocytes were harvested from the interface and washed twice in RPMI 1640 ready for activation of gamete formation. Blood cells from day 5 p.i mice were placed in culture (40 ml RPMI 1640, 8 ml foetal bovine serum, 0.5 ml penicillin and streptomycin; per 0.5 ml blood) for 24 h at 37°C for schizont- (with rotation at 100 rpm) and at 20°C for ookinete production, as described above. Schizonts and ookinetes were purified on 60% and 63% NycoDenz gradients, respectively and harvested from the interface and washed. For 14 dpi oocysts, 20 mosquito midguts were collected and homogenised with PBS in a loosely fitting homogeniser to release sporozoites as described above.

### Transcriptome sequencing and analysis

Parasites (activated gametocytes and ookinetes) were collected from *∆cyc3* or GFP-expressing lines. Samples were passed through a plasmodipur column to remove host DNA contamination prior to RNA isolation. Total RNA was isolated from purified parasites using an RNeasy purification kit (Qiagen). RNA was vacuum concentrated (SpeedVac) and transported using RNA stable tubes (Biomatrica). Strand-specific mRNA sequencing was performed from total RNA using TruSeq Stranded mRNA Sample Prep Kit LT (Illumina) according to the manufacturer's instructions. Briefly, polyA+ mRNA was purified from total RNA using oligo-dT dynabead selection. First strand cDNA was synthesised primed with random oligos followed by second strand synthesis where dUTPs were incorporated to achieve strand-specificity. The cDNA was adapter-ligated and the libraries amplified by PCR. Libraries were sequenced in an Illumina Hiseq machine with paired-end 100bp read chemistry.

RNA-seq read alignments were mapped onto the *P*. *berghei* ANKA genome (May 2015 release in GeneDB—http://www.genedb.org/) using Tophat2 (version 2.0.13) [89] with parameters “—library-type fr-firststrand–no-novel-juncs–r 60”. Transcript abundances (reported as FPKM- fragments per kilobase of exon per million fragments) were quantified and differential expression analysis was performed using Cuffdiff2 version 2.2.1 [90]. Genes with fold change greater than 2 and p-value less than 0.05 were considered as significantly differentially expressed. As a form of independent validation of the differentially expressed genes, transcript abundances were extracted as raw read counts using htseq-count [91] and differential expression analysis performed using DESeq2 [92] in R version 3.2.1. *P*. *berghei* GO terms (Gene Ontology) were downloaded from GeneDB (http://www.genedb.org/; May 2015 release) and gene ontology enrichment analysis was performed for the DEG lists using GOstats R package [93]. All analyses and visualizations were done with R packages- *cummeRbund* [94] and *ggplot2* [95].

### Quantitative RT-PCR

Total RNA was isolated from purified parasites using an RNeasy purification kit (Qiagen). For qRT-PCR, cDNA was synthesised using an RNA-to-cDNA kit (Applied Biosystems) allowing quantification from 250 ng of total RNA. qRT-PCR reactions consisted of 2 μl cDNA, 5 μl SYBR green fast master mix (Applied Biosystems), 0.5 μl (500 nM) each of the forward and reverse primers, and 2 μl DEPC-treated water. Where possible, one of the primer pairs was placed over an intron, primers had melting temperatures of 60–62°C and together amplified a region 70–200 bp long. Analysis was conducted using an Applied Biosystems 7500 fast machine with the following cycling conditions: 95°C for 20 sec followed by 40 cycles of 95°C for 3 sec; 60°C for 30 sec. Wild-type expression was determined using the Pfaffl method [96]. Relative quantification in the mutant line was normalised against wild-type expression using the ∆∆Ct method. Both methods used *hsp70* (and *arginine-tRNA synthetase* for wild-type expression) as a reference gene to provide a baseline of transcription levels between replicates to allow normalization of the data [29]. Three biological replicates were used for each stage (each with two technical replicates). See S1 Table for a full list of the primers used for qRT-PCR. Statistical analyses were performed using Excel and GraphPad Prism (GraphPad Software), with graphs showing normalised expression in ∆*cyc3* compared to a transcription baseline derived from WT. For relative gene expression, a Student’s unpaired *t*-test was used. For RNA-seq and qRT-PCR comparison, we used ≥30 genes at each stage and used GraphPad Prism (GraphPad Software) to calculate fit and coefficient of determination.

### Yeast experiments

Standard protocols of handling *Schizosaccharomyces pombe* and *Saccharomyces cerevisiae* were followed [97,98]. For yeast complementation experiments, the triple-*cln* mutant (*cln1∆ cln2∆ cln3∆ TRP*::*GAL1-CLN3 ade1 leu2-3 his2 trp1-1 ura3∆ bar1∆ pep4∆*::*LEU2*) or *cdc13 ts* mutant strain *(h- cdc13-117 leu1-32)* was used.

### Deconvolution microscopy

High resolution live cell imaging was performed using an Olympus-based Delta Vision Elite work station fitted with a 100x objective (numerical NA 1.4, oil). Post-acquisition analysis was carried out using Applied Precision software. Images presented are 2D projections of deconvolved Z-stacks of 0.3 μm optical sections.

### Ookinete motility assay

The ookinete motility assay was performed as previously described [99]. Twenty five microliters of the ookinete cultures were added to an equal volume of Matrigel (BD) on ice, mixed thoroughly, added to a slide, covered with a Vaseline-rimmed cover slip, and sealed with nail varnish. The Matrigel was then allowed to set at room temperature for at least 30 minutes. After identifying a field containing ookinetes, time-lapse videos (1 frame every 5 sec, for 10 min) were taken of ookinetes using the differential interference contrast (DIC) settings with a 63× objective lens on a Zeiss AxioImager M2 microscope fitted with an AxioCam ICc1 digital camera controlled by the Axiovision (Zeiss) software package. Speed of motility of individual ookinetes was measured by multiplying the number of body lengths moved by the length of the ookinete during the 10 min video, divided by 10. Multiple independent slides and cultures were used for each parasite line.

### Ookinete nuclear DNA content measurement

The nuclear content of the Δ*cyc3* ookinete was measured by the formula as previously described [58]. Briefly, to measure nuclear DNA content of microgametocytes, digital images of Hoechst-stained fixed or unfixed cells were obtained using a Zeiss AxioImager M2 microscope fitted with an AxioCam ICc1 digital camera and analysed using ImageJ software version 1.33u (National Institutes of Health, USA). The relative nuclear fluorescence intensity was determined with the following formula: Area (pixel) × (average intensity (relative units) − average background intensity (relative units)). The nuclear fluorescence intensity was standardized to the haploid DNA content by measuring the fluorescence intensity of ring-stage parasites in parallel on the same slide and with the same microscope and camera settings.

## Supporting Information

S1 Fig Phylogenetic analysis of cyclins. A maximum likelihood protein phylogeny comparing apicomplexan cyclins to sequences from human, *Schizosaccharomyces pombe*, and *Trypanosoma brucei*. Select *Arabidopsis* cyclins and Pho80 from *Saccharomyces cerevisiae* have been included for clarity of protein families. A consensus tree from 100 bootstrap replicates based on 270 alignable positions is shown with topology support at nodes. Protein domain architectures were predicted from the models in Pfam27 with e-value ≤ 0.001.
(TIF)

S2 Fig Generation and genotypic analysis of CYC3-GFP parasites. (A) Schematic representation of the endogenous *cyc3* locus, the GFP-tagging construct and the recombined *cyc3* locus following single cross-over recombination. Following recombination, the *cyc3* locus contains the tagged copy and the original *cyc3* CDS lacking all but 332 bp of upstream region, which is unlikely to sufficient for transcription. Arrows 1 and 2 indicate PCR primers used to confirm successful integration in the *cyc3* locus following recombination. (B) Integration PCR of the *cyc3* locus in wild type and CYC3-GFP parasites using primers IntT89 and ol492. Integration of *cyc3* with *gfp* gives a band of 1.2 kb. (C) Pulse Field Gel Electrophoresis (PFGE) using a *pbdhfr* 3’UTR probe. The probe recognises the endogenous *dhfr* locus on chromosome 7 and the recombined *cyc3* locus on chromosome 12. (D) Southern blot analysis of WT and *cyc3* parasite genomic DNA following BsmI digestion. A probe specific for the fragment homologous to the *P*. *berghei* genomic *cyc3* sequence cloned in the p277 vector bound to a 5.5 kb band in WT and to a 12 kb band in *Δcyc3* parasites. (E) Western blot of CYC3-GFP (54 kDa) and WT-GFP (29 kDa) protein to illustrate CYC3-GFP concentration in three different parasite stages. Total protein concentration for CYC3-GFP samples was normalised across all three samples and controlled by a Coomassie gel (see below the western blot). WT-GFP is shown as a control. Sch: schizont, AG: activated gametocytes, Ook: Ookinetes. (F) Total number of oocysts per infected mosquito at 14 dpi for CYC3-GFP and WT lines. Bar is the mean ± SEM. n = 2 independent experiments (15 mosquitoes for each) p>0.1. As the tagged line is not a clonal population, in the CYC3-GFP parasite line 86% of oocysts were expressing GFP. The rest of the oocysts are a WT population (not expressing GFP). (G) Individual CYC3-GFP and WT oocyst diameters measured in μm at 14 dpi p<0.001.
(TIF)

S3 Fig Generation and genotypic analysis of *∆cyc3* parasites. (A) Schematic representation of the endogenous *cyc3* locus, the targeting knock out construct and the recombined *cyc3* locus following double homologous cross-over recombination. Arrows 1 and 2 indicate PCR primers used to confirm successful integration in the *cyc3* locus following recombination and arrows 3 and 4 indicate PCR primers used to show deletion of the *cyc3* gene. (B) Integration PCR of the *cyc3* locus in WT and *∆cyc3* cl.1 and cl.3 parasites using primers INT N45 and ol248. Integration of the targeting construct gives a band of 0.7 kb. Presence of the gene gives a band of 0.2 kb. (C) Southern blot analysis of WT, *cyc3* cl.1 and *cyc3* cl.3 parasite genomic DNA following HindIII digestion. A probe specific for the *cyc3* 3’UTR bound to a 3.7 kb band in WT and to a 7.6 kb band in *∆cyc3* parasites. (D) Pulse Field Gel Electrophoresis (PFGE) using a *pbdhfr* 3’UTR probe. The probe recognises the endogenous *dhfr* locus on chromosome 7, the *gfp* cassette integrated in the 230p locus of the GFP-transgenic parasites used for transfection (chromosome 4) and the recombined *cyc3* locus on chromosome 12.
(TIF)

S4 Fig Sporozoite numbers in WT and ∆*cyc3* parasite lines and *∆cyc3* ookinetes show no observable phenotypes. (A) Total number of sporozoites per mosquito from 21 dpi salivary glands for *∆cyc3* and WT lines. Three independent experiments are described, n = 20 mosquitoes for each replicate. (B) Table of mosquito numbers for *∆cyc3* and WT lines. Bite back data are presented as day in which blood stage parasites are observed. dpi = days post infection. (C) Low power ultrastructural images of WT (i) and *Δcyc3* (ii) ookinetes. N–nucleus. M–micronemes. Bars represent 1 μm. (D) Graph representing the DNA content of Δ*cyc3* ookinetes compared to WT. (E) Graph representing the motility of Δ*cyc3* ookinetes compared to WT.
(TIF)

S5 Fig Complementation of cyclin in yeast. (A) *Plasmodium cyc3* cannot complement the triple *cln* (G1 cyclin) mutant of the budding yeast *Saccharomyces cerevisiae*. The triple-*cln* mutant *cln1 cln2 cln3* is lethal (+Glucose, empty vector), but it can be rescued by GAL-*CLN3* (+Galactose). *Plasmodium* cyc3, *CLN2* or empty vector was expressed under the control of the methionine-repressible MET3 promoter. In the absence of methionine (promoter *ON*), *Plasmodium* cyc3 (MET3-Pb_cyc3) was unable to rescue and form any colony whereas MET3-CLN2 rescued the triple *cln* mutant and these cells grew normally. (B) *Plasmodium cyc3* cannot complement the temperature-sensitive (ts) defect of a *cdc13-117* allele (B-type cyclin) of the fission yeast *Schizosaccharomyces pombe*. The ts *cdc13-117* mutant strains expressing the indicated plasmids were grown on the minimal medium plates in the absence of thiamine for 3 days at the restrictive temperature (36°C) or the permissive temperature (25°C). Cyclins were expressed from the *nmt1* medium-strength promoter (pREP41) or low-strength promoter (pREP81). Although *S*. *pombe cdc13* ^+^ rescued the temperature sensitivity (36°C), neither Pk epitope-tagged nor MH (c-myc and His6) tagged *Pb*cyc3 rescued. At the permissive temperature (25°C), all the strains grew normally.
(TIF)

S6 Fig Transcript analysis. (A) Strand-specific RNAseq reads aligned onto *Plasmodium berghei* genome, as visualized using Artemis. cyc3 gene is expressed in the wild types as shown by RNAseq reads in the reverse (-ve) strand, while a major portion of the gene is deleted in the knockouts as shown by the absence of RNAseq reads (area shaded in red).(B) Multidimensional scaling of gene expression values for WT and *∆cyc3* RNA-seq samples shows tight correlation among individual replicates within each sample group. (C) Heatmaps for cell cycle, inner membrane complex and apiAP2 transcription factor gene clusters based on their log_2_ fold change in *Δcyc3* activated gametocytes (inner circular track) and *Δcyc3* ookinetes (outer circular track) relative to WT. Functional groups were inferred from annotations available in GeneDB (http://www.genedb.org/). Genes that were found significantly misregulated are shown in bold and those validated by qRT-PCR are shown in red. Full gene list and functional clusters are shown in S3 Table. (D) GO term enrichment analysis of cyc3 activated gametocytes and ookinetes. The size of the dot is proportional to the level of significance and the color intensity represents the fold enrichment of enriched terms in biological process (green), molecular function (purple) and cellular component (pink).
(TIF)

S1 Table Oligonucleotides used in this study. (XLSX)

S2 Table List of differentially expressed genes (FDR-corrected p value < 0.05) between ∆*cyc3* and WT activated gametocytes and ookinetes. (XLSX)

S3 Table RNA-seq data and functional clusters used to generate the heat maps. (XLSX)
