# Supplementary figures and images for: Plasmodium P-Type Cyclin CYC3 Modulates Endomitotic Growth during Oocyst Development in Mosquitoes
Source: PLoS Pathog. 2015 Nov 13;11(11):e1005273. doi: 10.1371/journal.ppat.1005273 (PMC4643991; doi:10.1371/journal.ppat.1005273)

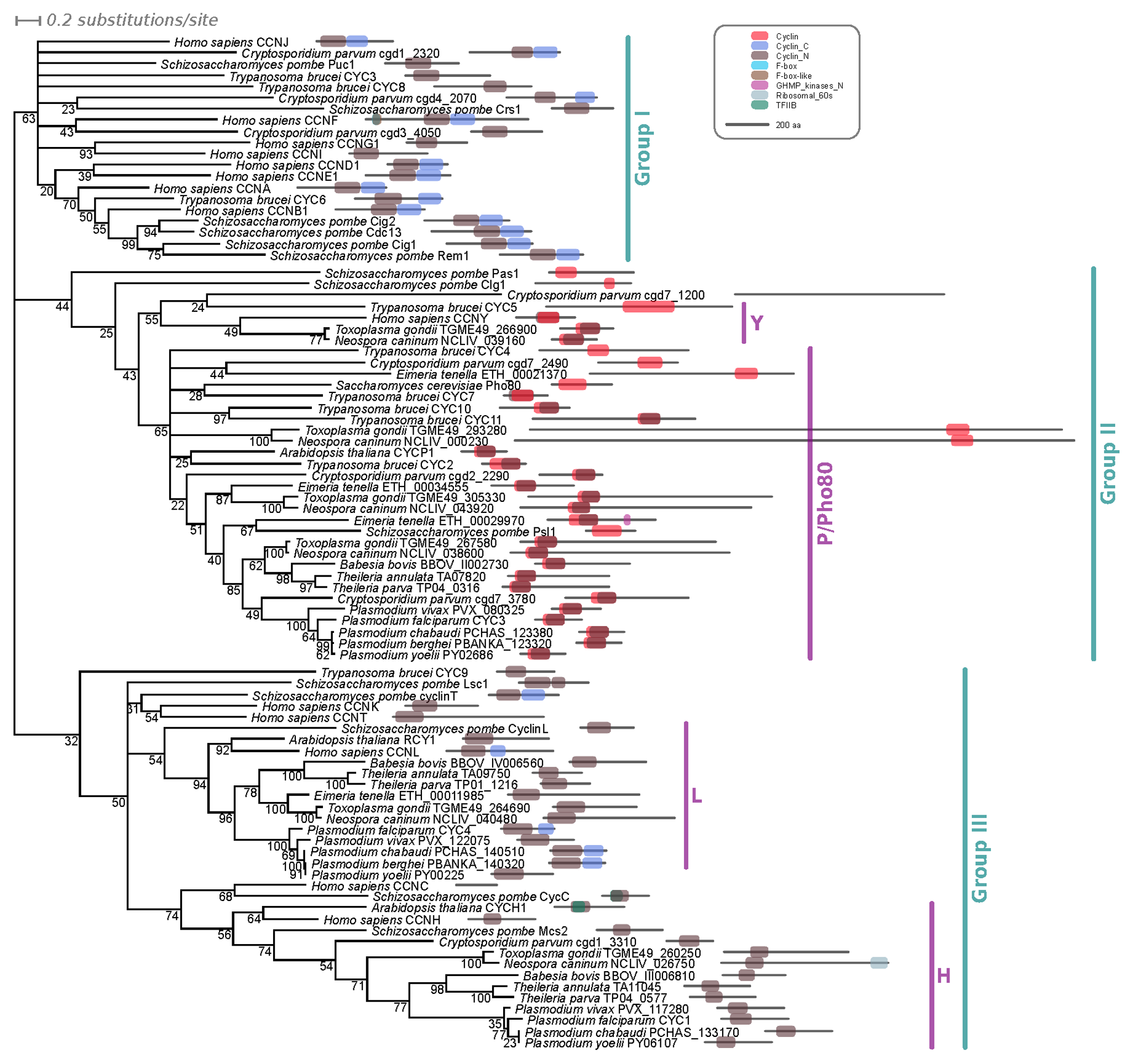

Supplement: S1 Fig — A maximum likelihood protein phylogeny comparing apicomplexan cyclins to sequences from human, Schizosaccharomyces pombe, and Trypanosoma brucei. Select Arabidopsis cyclins and Pho80 from Saccharomyces cerevisiae have been included for clarity of protein families. A consensus tree from 100 bootstrap replicates based on 270 alignable positions is shown with topology support at nodes. Protein domain architectures were predicted from the models in Pfam27 with e-value ≤ 0.001. (TIF) [file ppat.1005273.s001.tif]

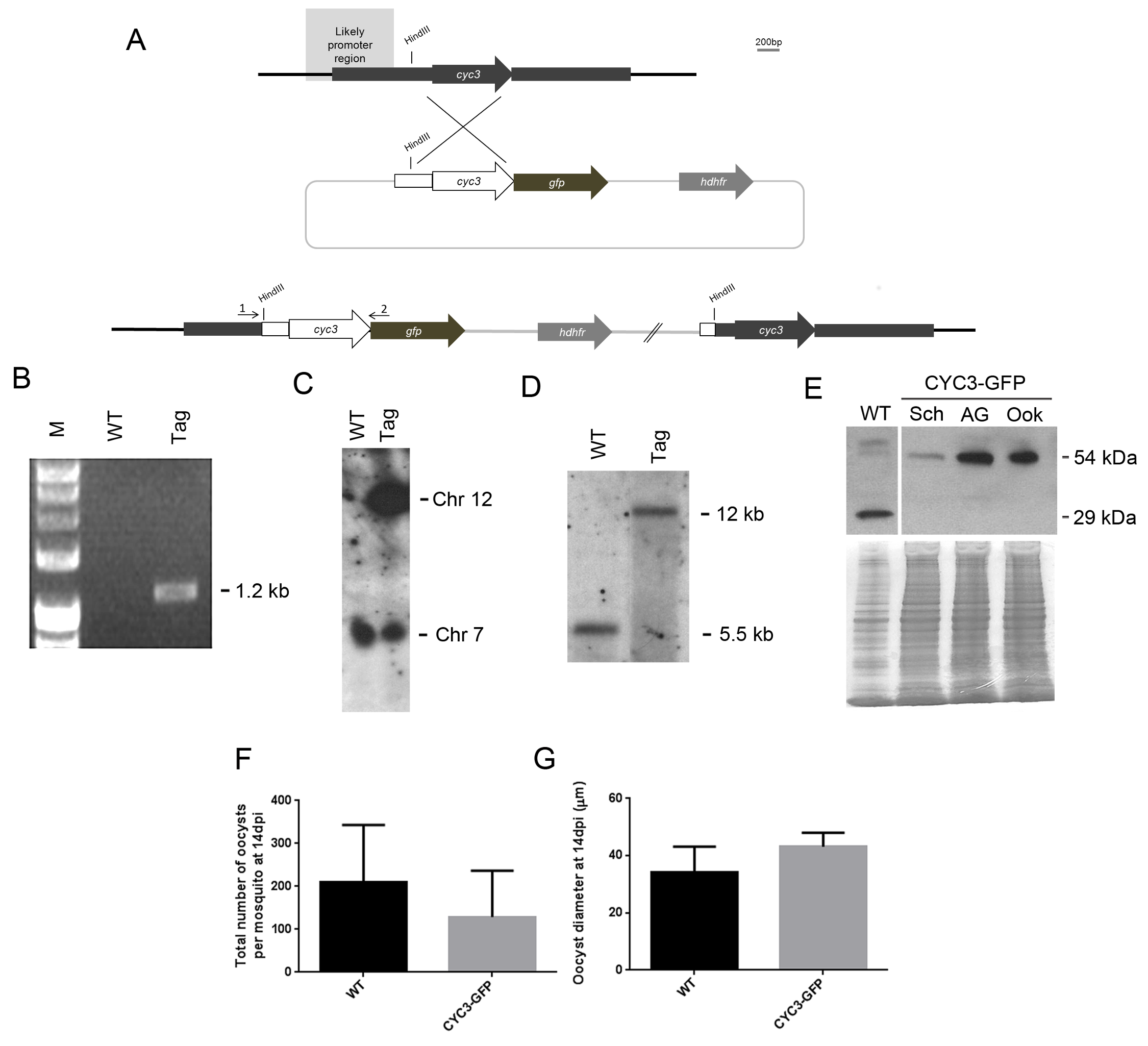

Supplement: S2 Fig — (A) Schematic representation of the endogenous cyc3 locus, the GFP-tagging construct and the recombined cyc3 locus following single cross-over recombination. Following recombination, the cyc3 locus contains the tagged copy and the original cyc3 CDS lacking all but 332 bp of upstream region, which is unlikely to sufficient for transcription. Arrows 1 and 2 indicate PCR primers used to confirm successful integration in the cyc3 locus following recombination. (B) Integration PCR of the cyc3 locus in wild type and CYC3-GFP parasites using primers IntT89 and ol492. Integration of cyc3 with gfp gives a band of 1.2 kb. (C) Pulse Field Gel Electrophoresis (PFGE) using a pbdhfr 3’UTR probe. The probe recognises the endogenous dhfr locus on chromosome 7 and the recombined cyc3 locus on chromosome 12. (D) Southern blot analysis of WT and cyc3 parasite genomic DNA following BsmI digestion. A probe specific for the fragment homologous to the P. berghei genomic cyc3 sequence cloned in the p277 vector bound to a 5.5 kb band in WT and to a 12 kb band in Δcyc3 parasites. (E) Western blot of CYC3-GFP (54 kDa) and WT-GFP (29 kDa) protein to illustrate CYC3-GFP concentration in three different parasite stages. Total protein concentration for CYC3-GFP samples was normalised across all three samples and controlled by a Coomassie gel (see below the western blot). WT-GFP is shown as a control. Sch: schizont, AG: activated gametocytes, Ook: Ookinetes. (F) Total number of oocysts per infected mosquito at 14 dpi for CYC3-GFP and WT lines. Bar is the mean ± SEM. n = 2 independent experiments (15 mosquitoes for each) p>0.1. As the tagged line is not a clonal population, in the CYC3-GFP parasite line 86% of oocysts were expressing GFP. The rest of the oocysts are a WT population (not expressing GFP). (G) Individual CYC3-GFP and WT oocyst diameters measured in μm at 14 dpi p<0.001. (TIF) [file ppat.1005273.s002.tif]

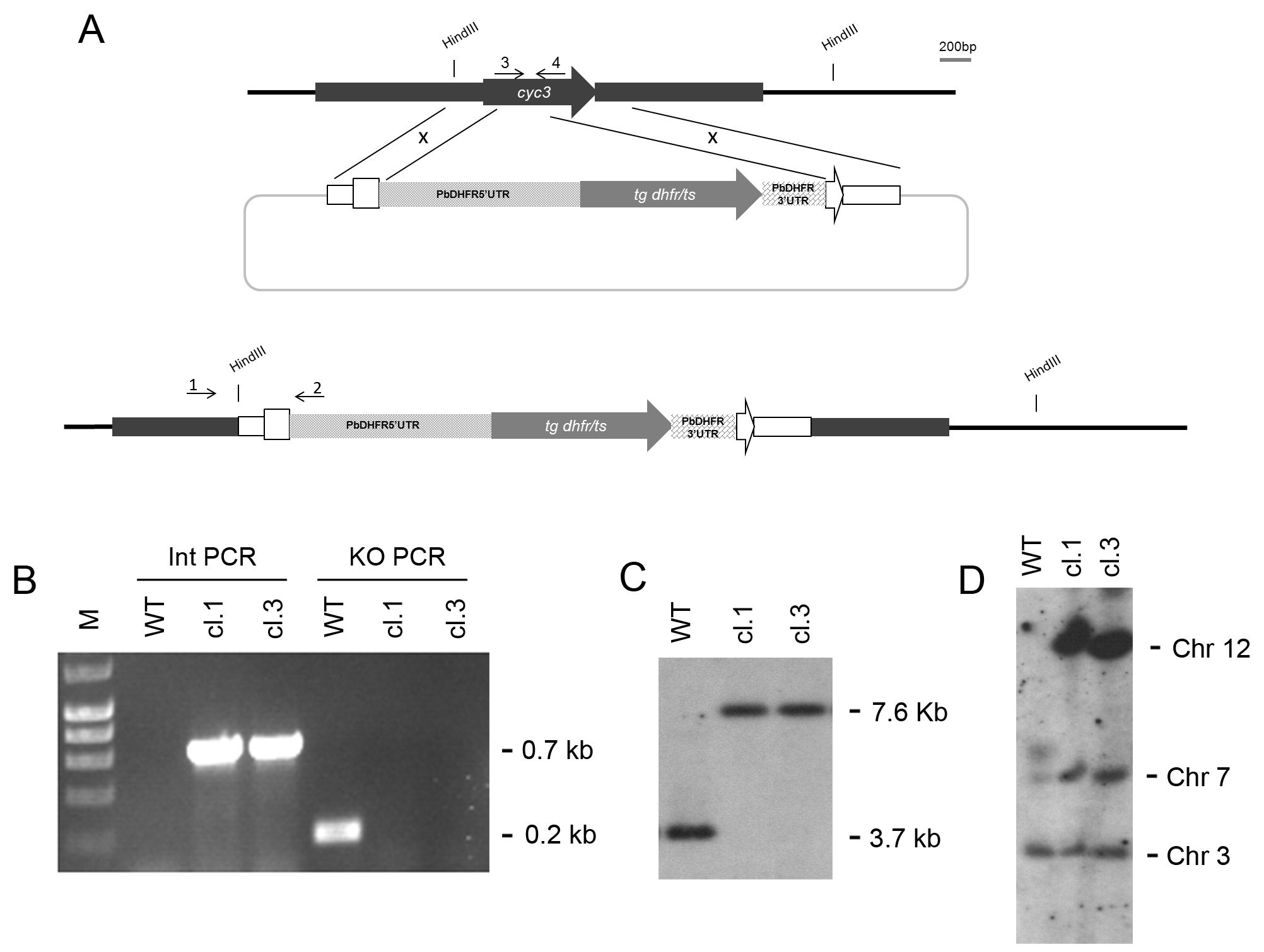

Supplement: S3 Fig — (A) Schematic representation of the endogenous cyc3 locus, the targeting knock out construct and the recombined cyc3 locus following double homologous cross-over recombination. Arrows 1 and 2 indicate PCR primers used to confirm successful integration in the cyc3 locus following recombination and arrows 3 and 4 indicate PCR primers used to show deletion of the cyc3 gene. (B) Integration PCR of the cyc3 locus in WT and ∆cyc3 cl.1 and cl.3 parasites using primers INT N45 and ol248. Integration of the targeting construct gives a band of 0.7 kb. Presence of the gene gives a band of 0.2 kb. (C) Southern blot analysis of WT, cyc3 cl.1 and cyc3 cl.3 parasite genomic DNA following HindIII digestion. A probe specific for the cyc3 3’UTR bound to a 3.7 kb band in WT and to a 7.6 kb band in ∆cyc3 parasites. (D) Pulse Field Gel Electrophoresis (PFGE) using a pbdhfr 3’UTR probe. The probe recognises the endogenous dhfr locus on chromosome 7, the gfp cassette integrated in the 230p locus of the GFP-transgenic parasites used for transfection (chromosome 4) and the recombined cyc3 locus on chromosome 12. (TIF) [file ppat.1005273.s003.tif]

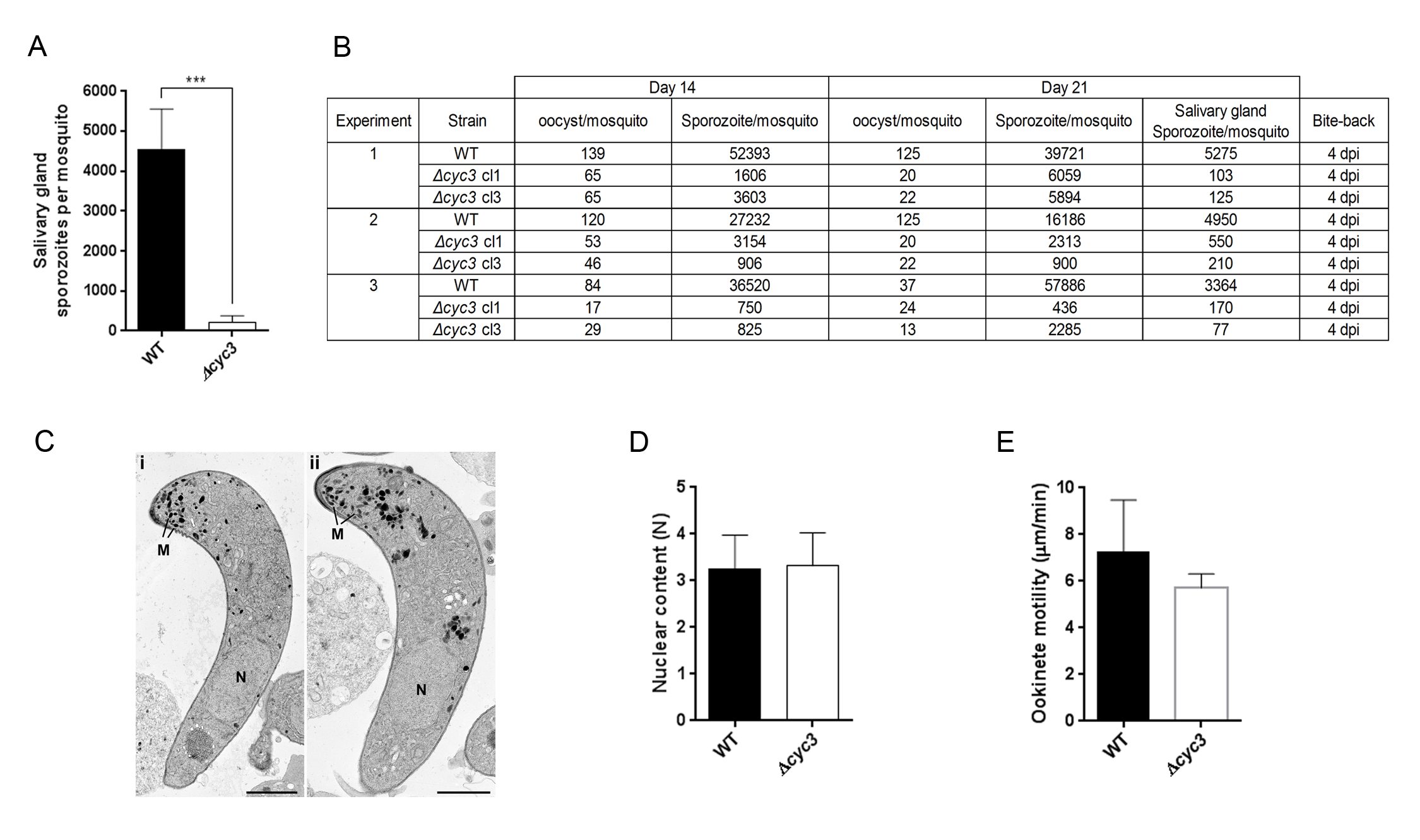

Supplement: S4 Fig — (A) Total number of sporozoites per mosquito from 21 dpi salivary glands for ∆cyc3 and WT lines. Three independent experiments are described, n = 20 mosquitoes for each replicate. (B) Table of mosquito numbers for ∆cyc3 and WT lines. Bite back data are presented as day in which blood stage parasites are observed. dpi = days post infection. (C) Low power ultrastructural images of WT (i) and Δcyc3 (ii) ookinetes. N–nucleus. M–micronemes. Bars represent 1 μm. (D) Graph representing the DNA content of Δcyc3 ookinetes compared to WT. (E) Graph representing the motility of Δcyc3 ookinetes compared to WT. (TIF) [file ppat.1005273.s004.tif]

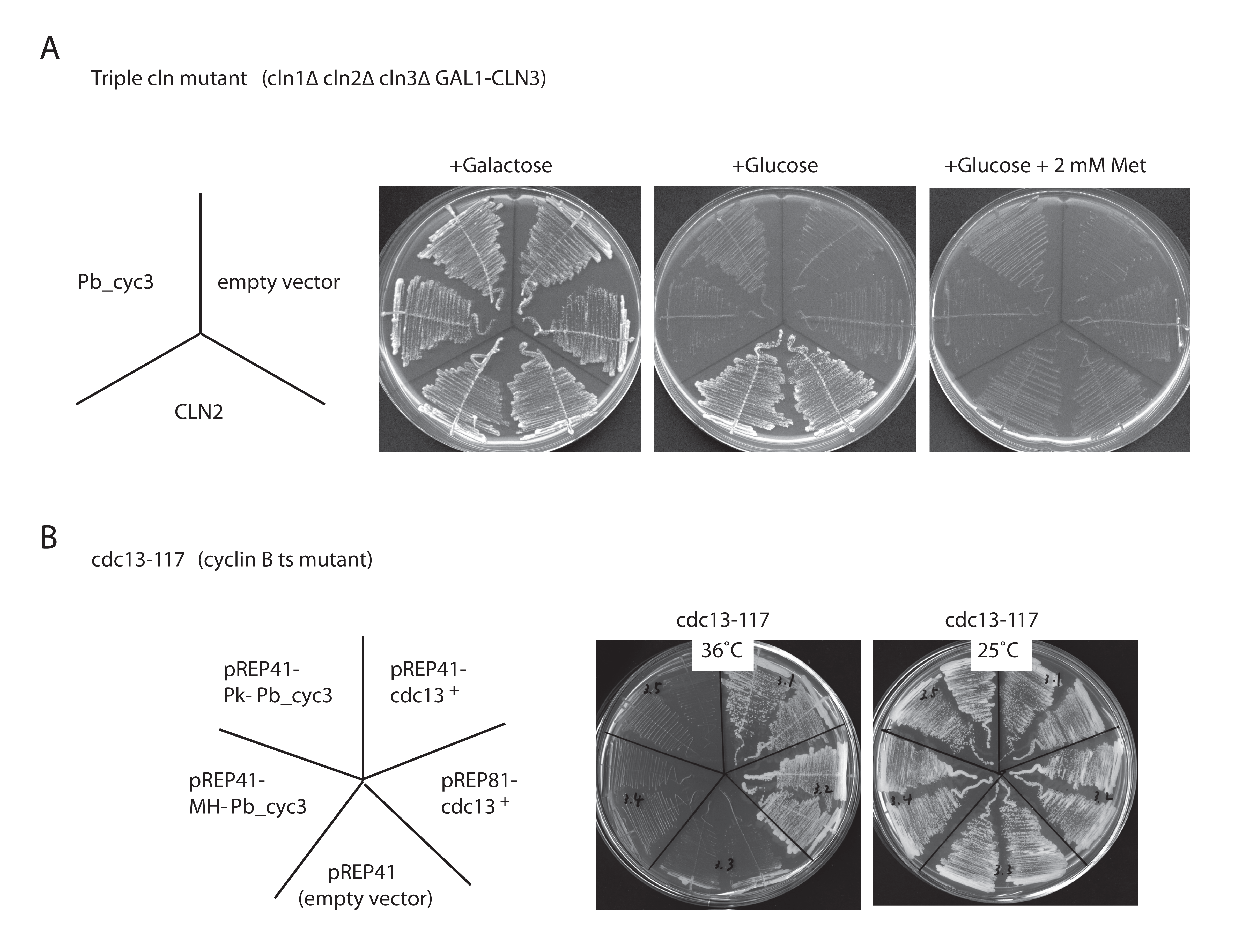

Supplement: S5 Fig — (A) Plasmodium cyc3 cannot complement the triple cln (G1 cyclin) mutant of the budding yeast Saccharomyces cerevisiae. The triple-cln mutant cln1 cln2 cln3 is lethal (+Glucose, empty vector), but it can be rescued by GAL-CLN3 (+Galactose). Plasmodium cyc3, CLN2 or empty vector was expressed under the control of the methionine-repressible MET3 promoter. In the absence of methionine (promoter ON), Plasmodium cyc3 (MET3-Pb_cyc3) was unable to rescue and form any colony whereas MET3-CLN2 rescued the triple cln mutant and these cells grew normally. (B) Plasmodium cyc3 cannot complement the temperature-sensitive (ts) defect of a cdc13-117 allele (B-type cyclin) of the fission yeast Schizosaccharomyces pombe. The ts cdc13-117 mutant strains expressing the indicated plasmids were grown on the minimal medium plates in the absence of thiamine for 3 days at the restrictive temperature (36°C) or the permissive temperature (25°C). Cyclins were expressed from the nmt1 medium-strength promoter (pREP41) or low-strength promoter (pREP81). Although S. pombe cdc13 + rescued the temperature sensitivity (36°C), neither Pk epitope-tagged nor MH (c-myc and His6) tagged Pbcyc3 rescued. At the permissive temperature (25°C), all the strains grew normally. (TIF) [file ppat.1005273.s005.tif]

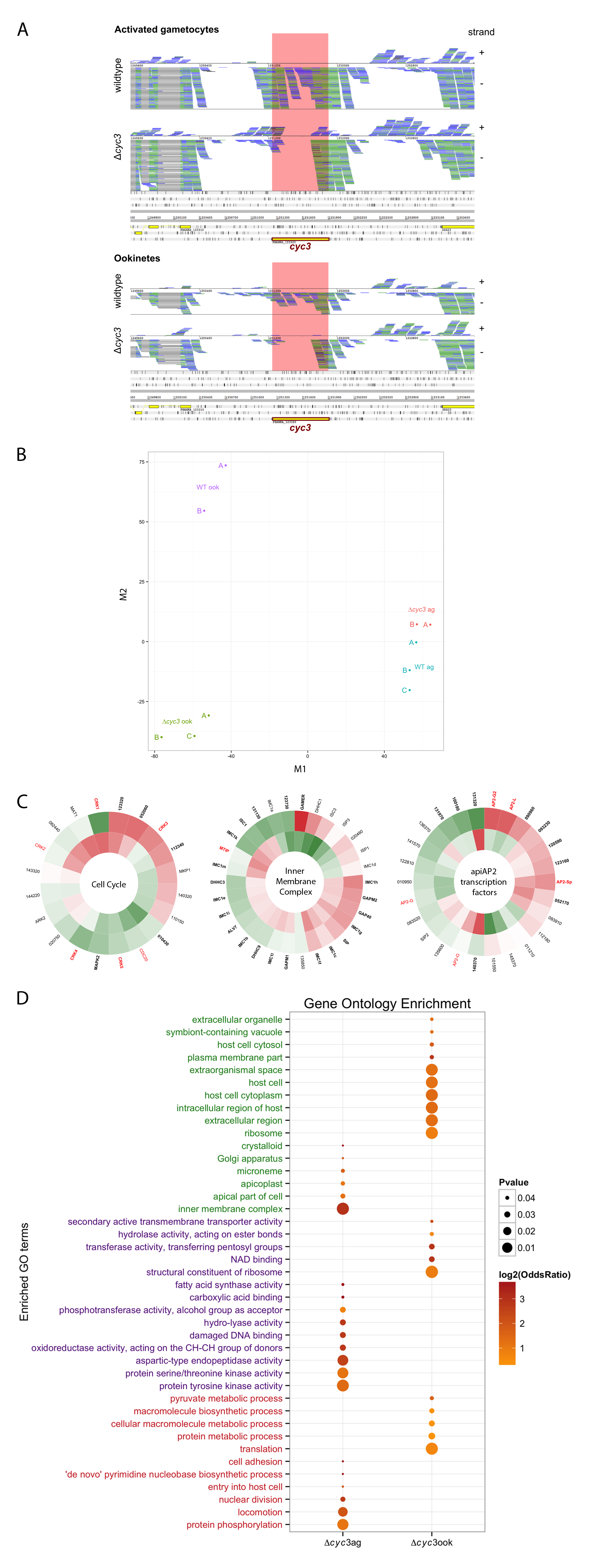

Supplement: S6 Fig — (A) Strand-specific RNAseq reads aligned onto Plasmodium berghei genome, as visualized using Artemis. cyc3 gene is expressed in the wild types as shown by RNAseq reads in the reverse (-ve) strand, while a major portion of the gene is deleted in the knockouts as shown by the absence of RNAseq reads (area shaded in red).(B) Multidimensional scaling of gene expression values for WT and ∆cyc3 RNA-seq samples shows tight correlation among individual replicates within each sample group. (C) Heatmaps for cell cycle, inner membrane complex and apiAP2 transcription factor gene clusters based on their log2 fold change in Δcyc3 activated gametocytes (inner circular track) and Δcyc3 ookinetes (outer circular track) relative to WT. Functional groups were inferred from annotations available in GeneDB (http://www.genedb.org/). Genes that were found significantly misregulated are shown in bold and those validated by qRT-PCR are shown in red. Full gene list and functional clusters are shown in S3 Table. (D) GO term enrichment analysis of cyc3 activated gametocytes and ookinetes. The size of the dot is proportional to the level of significance and the color intensity represents the fold enrichment of enriched terms in biological process (green), molecular function (purple) and cellular component (pink). (TIF) [file ppat.1005273.s006.tif]
